# Targeting CXCR4 abrogates resistance to trastuzumab by blocking cell cycle progression and synergizes with docetaxel in breast cancer treatment

**DOI:** 10.1186/s13058-023-01665-w

**Published:** 2023-06-06

**Authors:** Shuying Liu, Shelly M. Xie, Wenbin Liu, Mihai Gagea, Ariella B. Hanker, Nguyen Nguyen, Akshara Singareeka Raghavendra, Gloria Yang-Kolodji, Fuliang Chu, Sattva S. Neelapu, Adriano Marchese, Samir Hanash, Johann Zimmermann, Carlos L. Arteaga, Debasish Tripathy

**Affiliations:** 1 Department of Breast Medical Oncology, The University of Texas MD Anderson Cancer Center, Houston, TX USA; 2 Department of Bioinformatics and Computational Biology, The University of Texas MD Anderson Cancer Center, Houston, TX USA; 3 Department of Veterinary Medicine and Surgery, The University of Texas MD Anderson Cancer Center, Houston, TX USA; 4 Harold C. Simmons Comprehensive Cancer Center, The University of Texas Southwestern Medical Center, Dallas, TX USA; 5 Department of Internal Medicine, The University of Texas Southwestern Medical Center, Dallas, TX USA; 6 Department of Leukemia, The University of Texas MD Anderson Cancer Center, Houston, TX USA; 7 Department of Medicine, University of South California, Los Angeles, CA USA; 8 Department of Lymphoma-Myeloma, The University of Texas MD Anderson Cancer Center, Houston, TX USA; 9 Department of Biochemistry, Medical College of Wisconsin, Milwaukee, WI USA; 10 Department of Clinical Cancer Prevention, The University of Texas MD Anderson Cancer Center, Houston, TX USA; 11 Spexis Ltd, Allschwil, Switzerland

**Keywords:** Breast cancer, HER2, Drug resistance, CXCR4, Trastuzumab, Docetaxel, Targeted therapy, Combined therapy

## Abstract

**Background:**

Although trastuzumab and other HER2-targeted therapies have significantly improved survival in patients with HER2 overexpressed or amplified (HER2+) breast cancer, a significant proportion of patients do not respond or eventually develop clinical resistance. Strategies to reverse trastuzumab resistance remain a high clinical priority. We were the first to report the role of CXCR4 in trastuzumab resistance. The present study aims to explore the therapeutic potential of targeting CXCR4 and better understand the associated mechanisms.

**Methods:**

Immunofluorescent staining, confocal microscopy analysis, and immunoblotting were used to analyze CXCR4 expression. BrdU incorporation assays and flow cytometry were used to analyze dynamic CXCR4 expression. Three-dimensional co-culture (tumor cells/breast cancer-associated fibroblasts/human peripheral blood mononuclear cells) or antibody-dependent cellular cytotoxicity assay was used to mimic human tumor microenvironment, which is necessary for testing therapeutic effects of CXCR4 inhibitor or trastuzumab. The FDA-approved CXCR4 antagonist AMD3100, trastuzumab, and docetaxel chemotherapy were used to evaluate therapeutic efficacy in vitro and in vivo. Reverse phase protein array and immunoblotting were used to discern the associated molecular mechanisms.

**Results:**

Using a panel of cell lines and patient breast cancer samples, we confirmed CXCR4 drives trastuzumab resistance in HER2+ breast cancer and further demonstrated the increased CXCR4 expression in trastuzumab-resistant cells is associated with cell cycle progression with a peak in the G2/M phases. Blocking CXCR4 with AMD3100 inhibits cell proliferation by downregulating mediators of G2-M transition, leading to G2/M arrest and abnormal mitosis. Using a panel of trastuzumab-resistant cell lines and an in vivo established trastuzumab-resistant xenograft mouse model, we demonstrated that targeting CXCR4 with AMD3100 suppresses tumor growth in vitro and in vivo, and synergizes with docetaxel.

**Conclusions:**

Our findings support CXCR4 as a novel therapeutic target and a predictive biomarker for trastuzumab resistance in HER2+ breast cancer.

**Supplementary Information:**

The online version contains supplementary material available at 10.1186/s13058-023-01665-w.

## Background

Amplification of the human epidermal growth factor receptor 2 (HER2)/neu (ERBB2) gene and overexpression of the oncoprotein HER2 occur in around 20% of breast cancers, termed “HER2-positive (HER2+) breast cancer” [1–3]. The humanized anti-HER2 monoclonal antibody, trastuzumab (Herceptin), was the first oncogene-targeted therapy [4], and its use over the last 25 years has improved disease-free and overall survival in patients with early and advanced-stage HER2 + breast cancer [5–8]. However, resistance to trastuzumab remains a clinical challenge; many patients with advanced breast cancers do not respond or eventually develop clinical resistance. Reported underlying mechanisms of trastuzumab resistance include activation of the PI3K pathway [9], upregulation of the insulin-like growth factor-I receptor (IGF-1R) or an increase in IGF-1R/HER2 heterodimers [10, 11], and upregulation of epidermal growth factor receptor (EGFR), HER3/4, or their ligands [12–14], given that trastuzumab is unable to block ligand-induced EGFR/HER2 and HER2/HER3 heterodimers [15, 16]. In addition, variable HER2 C-terminal fragments are kinase-active but lack the trastuzumab-binding epitope [17]. Although many molecules/pathways have been implicated in trastuzumab resistance, the associated mechanisms remain unclear, and no biomarker can reliably predict a lack of benefit from trastuzumab. While several newer therapies have been approved for progressive HER2 + breast cancer, all rely on targeting HER2. Thus, further understanding the underlying mechanism of resistance to targeting HER2 is critical to improve outcomes for this breast cancer subtype.

In our previous studies, by establishing and using trastuzumab-resistant breast cancer cell models, we initially found upregulation of C-X-C motif chemokine receptor 4 (CXCR4), a G protein-coupled receptor (GPCR) of stromal cell-derived factor-1 (SDF-1α; CXCL12) in trastuzumab-resistant breast cancer. Knockdown of CXCR4 with shRNA sensitized the cells to trastuzumab [18–20]. Consistent with our findings, a very recent clinical study showed increased CXCR4 expression in trastuzumab-resistant breast cancer tissues and was associated with a higher risk of recurrence [21]. The SDF-1α/CXCR4 axis regulates the trafficking and homeostasis of immune cells and hematopoietic stem cells (HSCs) in bone marrow [22–24]. CXCR4 antagonist AMD3100 (plerixafor, Mozobil) is approved for use to mobilize HSCs to the peripheral blood for autologous transplantation [25]. In recent years, upregulation of CXCR4 has been found in many types of solid tumors [26–29]. CXCR4 signaling contributes to cancer metastasis [30–32] and suppresses anti-tumor immunity [33]. Given the impact of CXCR4 signaling on both tumor behavior and the immune system, its modulation may have cancer therapeutic implications.

In the present study, based on our previous findings of CXCR4 in trastuzumab resistance, we investigated the therapeutic potential of targeting CXCR4 in trastuzumab-resistant breast cancer models and explored the associated mechanisms.

## Materials and methods

### Drugs and reagents

AMD3100 and cisplatin were purchased from Selleckchem (Houston TX); trastuzumab, docetaxel, and carboplatin from MD Anderson Cancer Center Pharmacy; and recombinant human CXCL12/SDF-1α from R&D Systems (Minneapolis, MN).

### Cell culture and generation of stable cell lines

BT474 and SKBR3 cell lines were obtained from the American Type Culture Collection (Manassas, VA). All other cell lines were from the MDACC Characterized Cell Line Core at The University of Texas MD Anderson Cancer Center. Mycoplasma contamination and identity verification with short tandem repeat were performed regularly by the Core. BT474 and its derived cells were grown in Dulbecco modified Eagle medium/nutrient mixture/F-12 supplemented with 10% fetal bovine serum (FBS). SKBR3 and its derived cells were maintained in modified McCoy 5α, containing 1.5 mM l-glutamine, 2200 mg/l sodium bicarbonate, and 10% FBS. All other cells were cultured at Roswell Park Memorial Institute with 10% FBS. For establishment of trastuzumab-resistant cell lines, BT474 and SKBR3 cells were continuously exposed to trastuzumab (20 µg/ml) for at least 1 year. For generation of the cell lines stably knocking down CXCR4, cells were transduced with CXCR4 human shRNA lentiviral particles containing human short hairpin RNAs (shRNA; TL313630VC and TL313630VD; OriGene, Rockville, MD) [34, 35] according to the manufacturer’s protocol. Lentiviral particles containing non-effective scrambled shRNA provided by the manufacturer were used as a control. After transduction for 1 day, cells were selected with puromycin (1 µg/ml) for 2 weeks and pooled. BT474-derived trastuzumab-resistant HR6 cells were created in vivo by trastuzumab challenge for more than 1 month in athymic nude mice [12] and maintained in Improved Minimal Essential Medium (IMEM, Life Technologies, Inc.) supplemented with 10% FBS containing 10 ug/ml trastuzumab.

### Cell growth inhibition assay

Cell growth inhibition assays were performed in three-dimensional (3D) culture in Matrigel as previously described [36]. Briefly, 4 × 10^3^ cells were re-suspended in growth medium containing 2% growth factor-reduced Matrigel (BD Biosciences, NJ) and seeded in 8-well chambers coated with Matrigel (BD Biosciences). Drugs with SDF-1α (4 ng/ml) were added on day 5. The concentration of SDF-1α was used based on the circulating SDF-1α level in breast cancer patients. Medium with drugs was replaced every 3 days. Acini were photographed and counted in 10 randomly chosen fields. Total number and area of acini were quantitatively analyzed using AlphaView SA software (Cell Biosciences) and expressed as means ± standard deviation (SD), representative of three independent experiments.

### Clonogenic assay

Clonogenic assays were carried out as previously described [36]. Briefly, 700 cells were seeded in each well of 6-well plates in the growth medium for 14 days. For inhibitory assays, after attaching to the plate, the cells were treated with drugs and recombinant human SDF-1α (4 ng/ml) for 2 days. Then, the drugs were washed away, and the cells were allowed to grow in the growth medium for 18 days (HCC202, HCC1419, or their derived cells) or 14 days (BTRT and SKRT cells). After staining with 0.25% crystal violet in 20% ethanol, the total number and size of colonies were quantitatively analyzed using AlphaView SA software. Data were expressed as mean ± SD of triplicates, and results were representative of two independent experiments.

### Cell co-culture in 3D

Tumor cells, human breast cancer-associated fibroblasts (BCAF; Neuromics, MN), and human peripheral blood mononuclear cells (PBMC; Zen-Bio, CA) in 3D co-culture were performed as illustrated in Additional file 1: Fig. S1 or Fig. 2B. Spheres were photographed at the indicated time. At the end of the study, cell viability was quantitatively analyzed using the CellTiter-Glo 3D viability assay kit (Promega) following the manufacturer’s instructions. Relative luminescence units were measured using a microplate reader. Data were expressed as mean ± SD of triplicates, and results were representative of two independent experiments.

### In vitro antibody-dependent cellular cytotoxicity (ADCC) assay

In vitro ADCC assays were performed as described previously [37]. Briefly, human PBMCs were thawed following the protocol provided by the manufacturer. HCC1419-derived tumor cells were harvested and labeled with 5 (and 6)-carboxyfluorescein diacetate, succinimidyl ester (CFDA, SE; Molecular Probes, Inc). After washing, the labeled target cells were mixed with PBMCs at an effector: target ratio of 80:1. Trastuzumab was added to the mixed suspensions at a concentration of 100 µg/ml and incubated at 37 °C in a humidified 5% CO_2_ incubator. Ten hours later, the dead cells were stained with propidium iodide and analyzed using a Beckman Coulter Gallios flow cytometer.

### BrdU incorporation assay and dynamic CXCR4 detection

BTRT cells were seeded in the growth medium. After attaching, cells were serum starved for 24 h then FITC-BrdU pulse-labeled for 1 h. After washing, cells were cultured in growth medium until collection. For testing the effects of treatment with docetaxel on CXCR4 expression, cells were treated with docetaxel (5 nM) for 24 h before BrdU pulse and after BrdU pulse until cell collection. BrdU was detected with FITC-conjugated anti-BrdU antibody. CXCR4 was detected with the anti-human CXCR4 antibody MAB172 (R&D, Minneapolis, MN), with the IgG_2_B isotype (R&D) as a control, followed by staining with APC-conjugated secondary antibody (Invitrogen). Then cells were counterstained with 7-amino-actinomycin D (7-AAD) and analyzed using Beckman Coulter Gallios flow cytometer with Kaluza Analysis software.

### Cell cycle distribution

BTRT or SKRT cells were seeded in the growth medium containing 2% growth factor-reduced Matrigel and treated with vehicle or AMD3100 with the doses indicated on day 5. SDF-1α (4 ng/ml) was added at the same time. After treatment for 72 h, the cells were harvested and fixed in pre-cooled 70% ethanol. After incubation with RNase A and staining with propidium iodide (PI), the cells were analyzed by Beckman Coulter Gallios flow cytometer.

### Immunofluorescent staining and confocal microscopy analysis

For comparing CXCR4 expression in acquired trastuzumab-resistant cells or their parental cells, the cells were grown on coverslips pre-coated with polylysine and fixed in 4% paraformaldehyde for immunofluorescent staining. For testing the effect of AMD3100 on CXCR4 translocation induced by SDF-1α, the trastuzumab-resistant cells were treated with AMD3100 for 48 h. After serum starvation overnight, the cells received SDF-1α stimulation for the designed time. For immunofluorescent staining, cells were fixed in 4% paraformaldehyde at room temperature for 20 min and permeabilized in 0.25% Triton X-100 for 5 min. After blocking with 3% bovine serum albumin for 1 h, cells were incubated with the CXCR4 antibody overnight at 4 °C, followed by staining with Alexa Fluor 488-conjugated goat anti-mouse secondary antibody (Invitrogen). Nuclei were stained with 4′, 6-diamidino-2-phenylindole (DAPI; Thermo Scientific). After mounting, microscopic images were captured by a multiphoton confocal laser scanning microscope (Carl Zeiss, Thornwood, NY).

### Preparation of culture medium for breast cancer-associated fibroblasts and normal mammary fibroblasts

BCAFs or normal human mammary fibroblasts (HMF, Sciencell) were seeded in 96-well “U”-bottomed untouched plates at 10^4^ cells/well density and cultured in Dulbecco modified Eagle medium/nutrient mixture/F-12 with 10% FBS for 72 h. The medium was collected and centrifuged at a speed of 300 g for 10 min. The supernatant was collected and directly used for testing SDF-1α or stocked at − 80 °C.

### Enzyme-linked immunosorbent assay (ELISA)

SDF-1α in cell culture supernatant or in serum of breast cancer patients or similar-aged healthy women was quantified by ELISA using the Human CXCL12/SDF-1α Quantikine ELISA Kit (R&D Systems). Concentrations were calculated by comparing the sample absorbance to standard curves.

### Reverse phase protein array (RPPA)

Cells were seeded in 3D Matrigel and treated with AMD3100 (5 µM) and/or trastuzumab (20 µg/ml) starting on day 6 for 5 days. SDF-1α (4 ng/ml) was added at the same time. The cells were harvested from the Matrigel with pre-cooled 1X HBSS with 5 mM EDTA on ice and lysed in ice-cold lysis buffer [38]. The cell lysates were analyzed with RPPA [39, 40]. The antibodies used are listed in Additional file 9: Table S1. Human fresh-frozen tumor tissues were lysed in cold lysis buffer with homogenization and analyzed by RPPA [41].

### Western blot analysis

The treatment of the cells and preparation of cell lysis was the same as described in the RPPA subsection of “Materials and methods” section. Western blot analysis was performed as described previously [36]. Quantitative analysis of the bands was performed using AlphaView SA software.

### Immunohistochemical staining

Formalin-fixed paraffin-embedded (FFPE) tumor tissue sections were deparaffinized in xylene and then subjected to a gradient of alcohol, followed by retrieval in IHC-TekTM Epitope Retrieval Steamer with IHC-TekTM Epitope Retrieval Solution (IHC World) following the instruction of the manufacturer. To block endogenous peroxidase activity, the sections were incubated in 3% H_2_O_2_ for 10 min, followed by incubation in blocking buffer containing 2% horse serum, 1% BSA, 0.1% Triton X-100, 0.05% Tween 20 in PBS at room temperature for 30 min. Then sections were incubated with anti-CXCR4 antibody (1:400, Abcam) at 4 °C overnight. After washing, sections were incubated with goat anti-rabbit IgG-HRP (1:500, Abcam) for 1 h and visualized with DAB. The slides were analyzed with Keyence Microscope BZ-X810.

### Establishment of trastuzumab-resistant xenograft model and studies in vivo

Five-week-old female athymic nude mice (The Jackson Laboratory, Bar Harbor, ME) were implanted with 0.36-mg, 90-days release 17ꞵ-estradiol pellets (Innovative Research, Sarasota, FL). Three days later, 5 × 10^6^ HR6 cells [12] in 150 µl growth factor-reduced Matrigel and phosphate-buffered saline (1:1) were orthotopically injected. Once tumors reached a volume of ~ 100 mm^3^, the mice were randomly grouped and received treatment with vehicle, trastuzumab (20 mg/kg, intraperitoneally twice per week), AMD3100 (5 mg/kg, intraperitoneally twice per week), docetaxel (10 mg/kg, intraperitoneally once per week), or combinations as indicated. Tumor sizes were measured with calipers twice weekly. Tumor volume was calculated using the formula *V* = *lw*^2^/2. Differences in tumor volume between groups were analyzed using two-way ANOVA. At the end of the experiment, the mice were sacrificed with CO_2_. The tumors were harvested and subjected to double-blind histopathologic analysis by a veterinary pathologist.

### Human samples

Tumor and blood samples from breast cancer patients and healthy blood samples along with clinical data were obtained under protocols approved by the institutional review board at The University of Texas MD Anderson Cancer Center. Patients and tumor characteristics were collected by chart review [41]. The Institutional Review Board of MD Anderson approved the laboratory study. The tissues and serum samples were stored at 80 °C until further analysis.

### Statistical analyses

One-way ANOVA was used for multiple groups, and the *t*-test was used for two groups. Tumor growth curves were analyzed using two-way ANOVA, using Prism (GraphPad Software, La Jolla, CA). RPPA data were analyzed as previously described [39, 40] and followed by further analysis with one-way ANOVA to compare different groups. Data were expressed as mean ± SD. *P* values less than 0.05 were considered statistically significant.

## Results

### CXCR4 drives primary trastuzumab resistance in HER2 + breast cancer, and pharmacologic inhibition of CXCR4 sensitizes the cells to trastuzumab

**Fig. 1:**
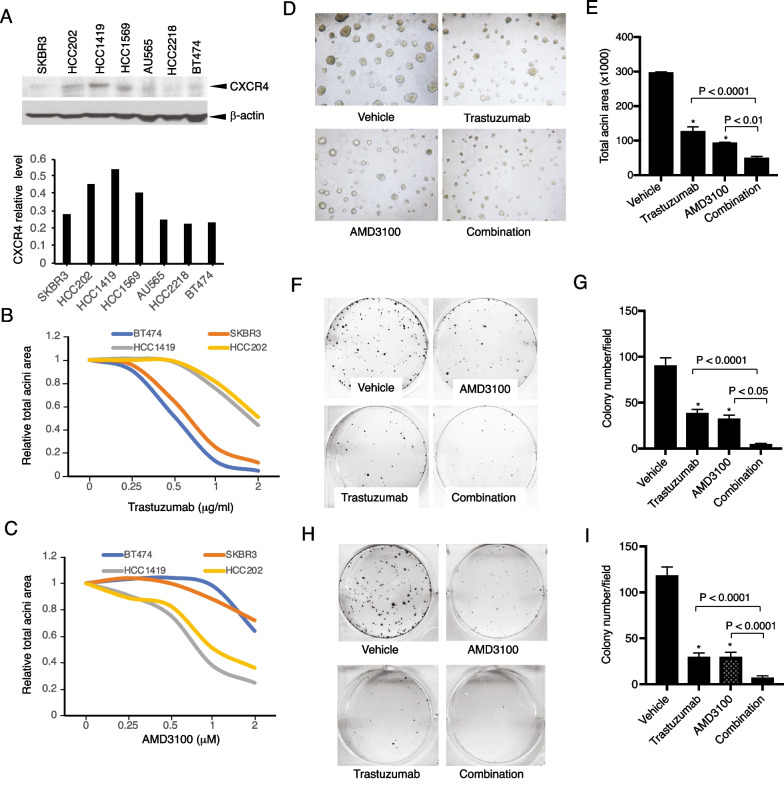
Targeting CXCR4 abrogates trastuzumab resistance. **A** HER2 + breast cancer cells with or without primary trastuzumab resistance were examined for CXCR4 expression with its antibody (UMB2, Abcam) using western blot analysis. The density of the bands was quantitatively analyzed. **B**, **C** Cell lines with high expression of CXCR4 (HCC1419 and HCC202) and low expression of CXCR4 (BT474 and SKBR3) were seeded in 3D Matrigel and treated with trastuzumab (**B**) or AMD3100 with SDF-1α (4 ng/ml). (**C**). The total area of the acini was quantitatively analyzed (see “Materials and methods” section). **D** HCC1419 cells grown in 3D Matrigel culture were treated with trastuzumab (2 µg/ml), AMD3100 (1 µM), SDF-1α (4 ng/ml), or the combination. Photographs were taken on day 13 after the start of treatment. The total area of the acini was quantitatively analyzed using AlphaView SA software (**E**). **F**–**I** Clonogenic assay. HCC1419 (**F**) and HCC202 (**H**) cells were seeded at low density and treated with AMD3100 (0.5 µM), SDF-1α (4 ng/ml), trastuzumab (2.5 µg/ml), or the combination. The plates were scanned on day 18 after the start of treatment. Colony formation was quantitatively analyzed using AlphaView SA software. **E**, **G**, **I** Data were analyzed using one-way ANOVA and are reported as mean ± SD of triplicates, representing two independent experiments (*P < 0.0001 compared with vehicle)

To confirm that CXCR4 contributes to trastuzumab resistance, we analyzed CXCR4 protein expression in a panel of HER2 + human breast cancer cell lines that were confirmed with different sensitivities to trastuzumab [42]. Compared with the trastuzumab-sensitive cell lines, the trastuzumab-resistant cell lines exhibited higher CXCR4 expression (Fig. 1A). To investigate the functional role of CXCR4, we used cell lines with high CXCR4 expression (CXCR4-high; HCC1419, HCC202) and low CXCR4 expression (CXCR4-low; BT474, SKBR3) for further studies. Cells were treated with serial concentrations of trastuzumab in 3D Matrigel culture. CXCR4-high cells showed higher tolerance to trastuzumab than CXCR4-low cells (Fig. 1B). Trastuzumab-resistant cells exhibited more sensitivity to the CXCR4 antagonist AMD3100 (Fig. 1C). The combination of AMD3100 and trastuzumab in CXCR4-high HCC1419 cells significantly increased the inhibitory effects on acini growth than either monotherapy (*P* < 0.0001 compared with trastuzumab alone, *P* < 0.01 compared with AMD3100 alone; Fig. 1D, E). We also investigated the role of CXCR4 in cell survival using clonogenic assays. AMD3100 or trastuzumab each individually inhibited colony formation (*P* < 0.0001 compared with the vehicle). However, the combined treatment had markedly greater inhibitory effects than either drug alone in HCC1419 cells (*P* < 0.0001 compared with trastuzumab monotherapy, *P* < 0.05 compared with AMD3100 monotherapy; Fig. 1F, G) and HCC202 cells (both *P* < 0.0001 compared with each monotherapy; Fig. 1H, I). These results suggest that CXCR4 contributes to primary resistance to trastuzumab, and inhibition of CXCR4 sensitizes the cells to trastuzumab.

### Knockdown of CXCR4 abrogates trastuzumab resistance in HER2 + breast cancer cells

**Fig. 2:**
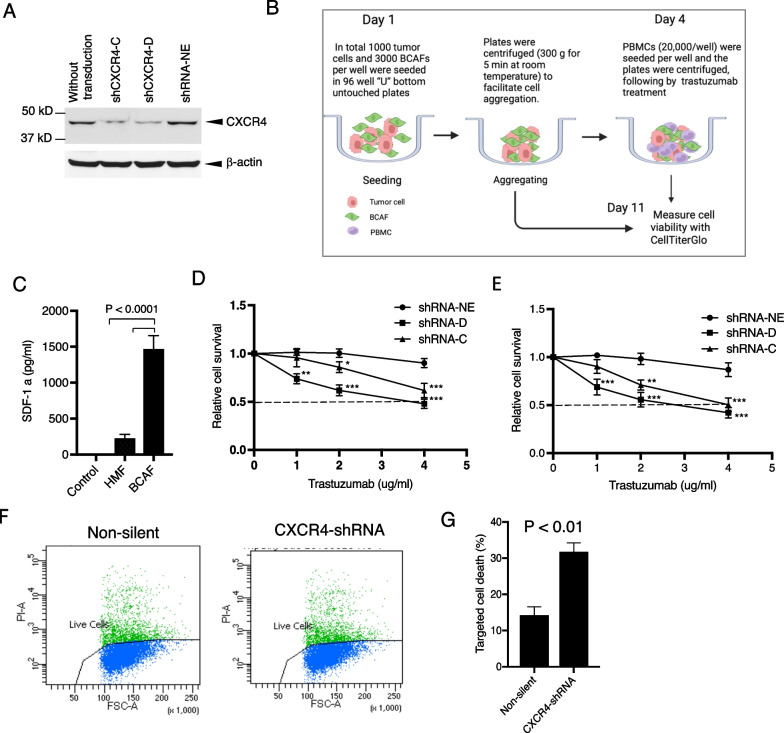
Knockdown of CXCR4 abrogates trastuzumab resistance. CXCR4 was silenced by specific shRNA in HCC1419 cells (“Materials and methods” section). The puromycin-resistant stable colonies were pooled together and named shCXCR4. A pool of cells infected with the lentivirus containing a non-effective vector (shRNA-NE) was selected and used as the control. **A** Western blot analysis was used to confirm the reduction in CXCR4 expression. **B** Illustration of co-cultures. **C.** BCAFs and HMFs were cultured in Dulbecco modified Eagle medium/nutrient mixture/F-12 supplemented with 10% FBS for 72 h. The culture supernatant was collected and tested for SDF-1α using ELISA following the manufacturer’s instructions. The medium used for the cell culture was used as the negative control. The data were analyzed with one-way ANOVA. **D**, **E** CXCR4-knockdown cells or non-silent control cells were co-cultured with BCAFs (**D**) or with BCAFs and PBMCs (**E**) in 3D, followed by treatment with trastuzumab as illustrated in **B**. At the endpoint of the study, relative cell viability was quantitatively analyzed using CellTiter-Glo 3D viability assay kit. The data were analyzed with one-way ANOVA (*P < 0.05, **P < 0.01, ***P < 0.001 compared with the non-silent control cells). **F**, **G** CXCR4-knockdown cells or non-silent control cells were used for trastuzumab-induced antibody-dependent cellular cytotoxicity (detail in “Materials and methods” section). The cells were stained with propidium iodide and analyzed by flow cytometry (**F**). Data were analyzed using *t*-test analysis of variance and are reported as the mean ± SD of triplicates (**G**)

To further confirm the contribution of CXCR4 to trastuzumab resistance, we silenced CXCR4 using specific shRNA in HCC1419 cells with primary trastuzumab resistance (see “Materials and methods” section for details). Reduction of CXCR4 expression in the puromycin-resistant stable cell lines was confirmed (Fig. 2A). Trastuzumab mediates tumor regression by interrupting HER2 oncogenic signals and is also able to induce Fcγ receptor-mediated ADCC mainly mediated by natural killer cells [43, 44]. To mimic the tumor microenvironment, we performed tumor cells/BCAFs (two-line) or tumor cells/BCAFs/PBMCs (three-line) co-cultures as illustrated in Fig. 2B. SDF-1α production by BCAFs was detected with ELISA. Comparing normal human mammary fibroblasts, BCAFs produced more SDF-1α (Fig. 2C). In the two-line co-culture system, breast cancer tumor cells with or without CXCR4-knockdown express different levels of CXCR4, while BCAFs produce SDF-1α, which allowed us to test the effect of knockdown of CXCR4 on the function of trastuzumab by interrupting HER2 oncogenic signals. In the three-line co-culture system, using tumor cells with different CXCR4 expression levels as the target cells, and PBMCs as the effector cells, allowed us to test the effect of knockdown of CXCR4 on the function of trastuzumab-mediated tumor regression by both interrupting HER2 oncogenic signals and ADCC. Cell viability was quantitatively analyzed (see “Materials and methods” section). Knockdown of CXCR4 sensitized the tumor cells to trastuzumab in two-line co-culture (Fig. 2D), suggesting CXCR4 plays a role in the function of trastuzumab by interrupting HER2 oncogenic signals. Comparing the two-line co-culture, adding PBMCs (three-line co-culture) shows mildly increased tumor cell sensitivity to trastuzumab (Fig. 2E).

We also performed trastuzumab-induced ADCC assays with flow cytometry analysis [37] (“Materials and methods” section, in detail). The HCC1419-derived cells were used as target cells, and the PBMCs were used as the effector cells. Consistent with the three-line co-culture above, CXCR4-knockdown cells exhibited an augmented response to trastuzumab (*P* < 0.01; Fig. 2F, G).

Taken together, these findings showed that CXCR4 plays a role in primary resistance to trastuzumab in HER2 + breast cancer, and combined targeting of CXCR4 sensitizes the tumor cells to trastuzumab.

### Continuous trastuzumab challenge induces acquired drug resistance and upregulation of CXCR4

**Fig. 3:**
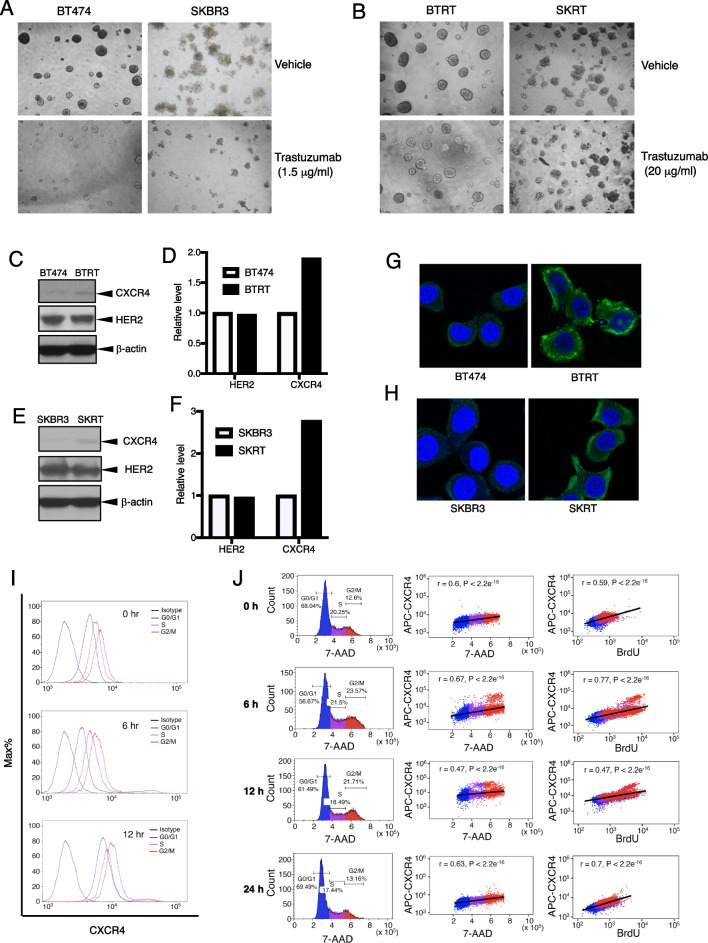
Creation of acquired trastuzumab-resistant cell models and characteristics of CXCR4 expression. To create cell models of acquired trastuzumab resistance, we continuously exposed BT474 and SKBR3 cells to trastuzumab (20 µg/ml) for at least 1 year. Tolerances to trastuzumab of primary cells (**A**) or the trastuzumab-resistant cells (**B**) were tested in 3D Matrigel culture. Photographs were taken on day 13. Expression of CXCR4 and HER2 was evaluated in BTRT (**C**) and SKRT (**E**) cells. Quantitative analysis of the density was performed using AlphaView SA software (**D**, **F**). CXCR4 expression in BTRT (**G**) and SKRT (**H**) cells was verified with immunofluorescent staining (green) and examined under a confocal microscope (see “Materials and methods” section). **I** Dynamic expression of CXCR4 with cell cycle progression in BTRT cells was detected by flow cytometry. **J** BTRT cells received BrdU pulse. Immunofluorescent staining for CXCR4, BrdU, and 7-AAD was performed and followed by flow cytometry analysis (“Materials and methods” section). The correlation between CXCR4 and 7-AAD or BrdU was analyzed using Pearson r coefficients

To confirm that CXCR4 plays a role in acquired trastuzumab resistance, we re-created trastuzumab-resistant breast cancer models via continuous exposure of the trastuzumab-sensitive cells to trastuzumab (20 µg/ml) for at least 1 year. BT474 and SKBR3 cell lines were used to represent HER2+/estrogen receptor (ER) + and HER2 + /ER- breast cancer, respectively. The cells that acquired trastuzumab resistance were designated as BTRT and SKRT, respectively. Drug resistance was verified in the cells. Trastuzumab at a low concentration (1.5 µg/ml) markedly inhibited the primary cell growth in 3D Matrigel culture (Fig. 3A). As expected, the cells with acquired trastuzumab resistance exhibited tolerance to trastuzumab at much higher concentration (20 µg/ml; Fig. 3B). Upregulation of CXCR4 protein was found in both BTRT cells (Fig. 3C, D) and SKRT cells (Fig. 3E, F) compared with BT474 and SKBR3 cells, respectively, whereas HER2 expression did not change significantly after acquired trastuzumab resistance. Consistent with the western blot analysis results, immunofluorescence staining showed overexpression of CXCR4 in BTRT (Fig. 3G) and SKRT cells (Fig. 3H). These results indicate that CXCR4 upregulation is associated with acquired trastuzumab resistance.

### CXCR4 expression increases with cell cycle progression and reaches a peak in the G2/M phases

We next investigated the dynamic expression of CXCR4 in acquired trastuzumab-resistant cells with the BrdU assay, in which BrdU was incorporated into newly synthesized DNA and stained with the FITC-conjugated anti-BrdU antibody; total DNA was detected with 7-amino-actinomycin D (7-AAD) and a specific primary antibody for CXCR4 and an APC-conjugated secondary antibody were used to detect CXCR4 (“Materials and methods” in detail). Three-color flow cytometry analysis permits testing CXCR4 expression in different phases of the cell cycle. CXCR4 expression steadily increased from G0/G1 phase to S phase and reached the highest level in the G2/M phases (Fig. 3I). Pearson correlation coefficient analysis showed a high positive coefficient between CXCR4 expression and total DNA content, the two continuous variables (Fig. 3J, middle panel). Results at 6 h and 12 h after BrdU pulse showed higher CXCR4 expression in newly divided BrdU-positive cells than in relatively aged BrdU-negative cells. CXCR4 expression returned to baseline 24 h later (Fig. 3J, right panel). The dynamic variation of CXCR4 supports that CXCR4 expression is associated with cell cycle progression in trastuzumab-resistant breast cancer cells.

### Inhibition of CXCR4 reverses the aggressive behavior of breast cancer cells with acquired trastuzumab resistance

**Fig. 4:**
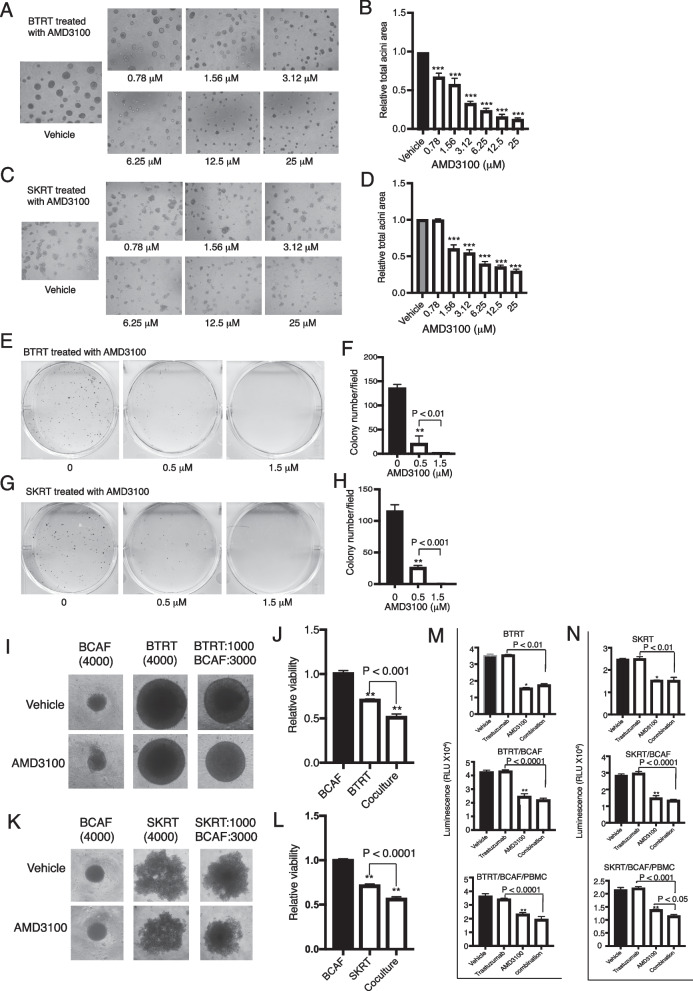
CXCR4 antagonist inhibits aggressive behavior in HER2 + breast cancer cells with acquired trastuzumab resistance. BTRT (**A**) and SKRT (**C**) cells were grown in 3D Matrigel followed by treatment with serial doses of AMD3100 (“Materials and methods” section). Photographs were taken on day 13. The total acini area was quantitatively analyzed with AlphaView SA. The data were analyzed using one-way ANOVA (**B**, **D**). BTRT (**E**) and SKRT (**G**) cells were seeded at low density and treated with different doses of AMD3100. The plates were scanned on day 18. Colony numbers were quantitatively analyzed using AlphaView SA. The data were analyzed using one-way ANOVA (**F**, **H**). BTRT (**I**) and SKRT (**K**) cells were co-cultured with BCAFs in 96-well “U”-bottomed unattached plates and treated with AMD3100 (2.5 µM; “Materials and methods” section). Dynamic changes of the spheres were monitored and photographed. At the end of the study, viability of the cells in monoculture or co-culture was detected using CellTiter-Glo 3D viability assay kit. The cell viability ratio of treated with AMD3100 to vehicle was analyzed using one-way ANOVA (**J**, **L**). BTRT (**M**) and SKRT (**N**) cells were co-cultured with BCAFs (two lines) or with BCAFs and PBMCs (three lines), followed by treatment with trastuzumab (20 µg/ml) and/or AMD3100 (2.5 µM) as illustrated in Fig. S1. At the endpoint, cell viability was detected using CellTiter-Glo 3D viability assay kit and analyzed using one-way ANOVA. The data are reported as mean ± SD of triplicates, representing two independent experiments (*P < 0.01, **P < 0.001, ***P < 0.0001 compared with vehicle). Recombinant SDF-1α (4 ng/ml) was added in all the experiments for testing AMD3100 effect but the co-culture studies with BCAFs that produce SDF-1α

To investigate whether targeting the cell cycle progression-associated CXCR4 affects cell proliferation, we seeded BTRT and SKRT cells in Matrigel and treated the cells with AMD3100. AMD3100 dose-dependently inhibited acini growth of BTRT (Fig. 4A, B) and SKRT (Fig. 4C, D) cells (*P* < 0.0001 compared with vehicle). We also tested the effect of AMD3100 on cell survival using clonogenic assays. With a similar pattern to that exhibited in cell growth assays, AMD3100 dose-dependently inhibited colony formation in BTRT (Fig. 4E, F) and SKRT (Fig. 4G, H) cells (*P* < 0.0001 compared with vehicle).

To mimic the microenvironment of breast cancer, we again co-cultured trastuzumab-resistant HER2 + breast cancer cells with BCAFs followed by treatment with or without AMD3100. The monocultures were used as controls. Spheres were photographed every 4 days. Compared with vehicle, AMD3100 inhibited growth of the spheres formed by BTRT cells, but not those formed by BCAFs. However, the inhibitory effect was further increased in the co-culture of BTRT and BCAFs (Fig. 4I; Additional file 2: Fig. S2). Co-cultures of SKRT with BCAFs showed similar results (Fig. 4K; Additional file 2: Fig. S2). Cell viability was quantitatively analyzed at the end of the study (“Materials and methods” in detail). Consistent with the size of spheres, AMD3100 inhibited the viability of BTRT and SKRT cells in monoculture (*P* < 0.0001). The inhibitory effect was further increased in the co-cultures of BTRT cells and BCAFs (*P* < 0.001; Fig. 4J) and SKRT cells and BCAFs (*P* < 0.0001; Fig. 4L) but did not affect the viability of BCAFs compared with vehicle.

Because growing evidence suggests that trastuzumab requires the engagement of the immune system for effectiveness [43, 44], we further co-cultured the tumor cells and BCAFs with or without PBMCs. The spheres were treated with AMD3100, trastuzumab, or the combination (Additional file 1: Fig. S1). AMD3100 inhibited tumor cell growth in monoculture (*P* < 0.001) and co-culture (*P* < 0.0001). Adding trastuzumab to AMD3100 did not further increase the efficacy in BTRT monoculture, but mildly increased the inhibitory efficacy in co-cultures, particularly with immune engagement (Fig. 4M). As expected, trastuzumab alone did not inhibit viability of the tumor cells with acquired trastuzumab resistance in monoculture or co-cultures with BCAFs and/or PBMCs. A similar pattern was observed in SKRT cells (Fig. 4N).

Taken together, these results indicate that CXCR4 contributes to acquired trastuzumab resistance, and targeting CXCR4 with its antagonist reverses resistance.

### Targeting CXCR4 with AMD3100 restrains cell division by inhibiting mediators of G2-M transition and mitosis

**Fig. 5:**
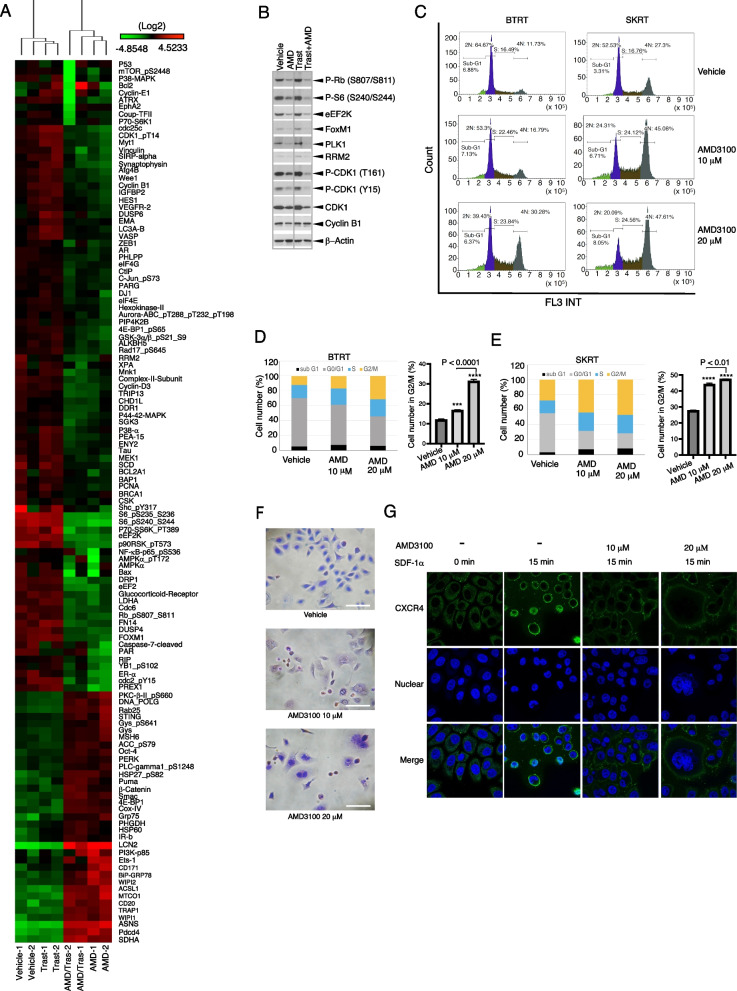
Mechanism of AMD3100 function in HER2 + breast cancer with trastuzumab resistance. **A** BTRT cells grown in 3D Matrigel culture were treated with vehicle, AMD3100 (10 µM), trastuzumab (20 µg/ml), or their combination. SDF-1α (4 ng/ml) was added at the same time. Cell lysates were subject to RPPA (“Materials and methods” section). Data are presented in a matrix format: each row represents an antibody target and each column a sample. In each sample, the ratio of the abundance of the molecule to its median abundance across all samples is represented by the color of the corresponding cell in the matrix (see the scale for expression levels). **B** The cells were treated the same as **A**. Cell lysates were subjected to western blot analysis. **C** BTRT and SKRT cells grown in 3D Matrigel culture were treated with AMD3100 or vehicle for 3 days. SDF-1α (4 ng/ml) was added at the same time. The cells were collected from the Matrigel and analyzed to determine the phases of the cell cycle using flow cytometry. **D**, **E** Quantitative analysis was performed, and the data were analyzed using one-way ANOVA. The data are reported as mean ± SD of triplicates, representing two independent experiments (*P < 0.001, **P < 0.0001 compared with vehicle). **F** SKRT cells grown on coverslips pre-coated with poly-l-lysine were treated with AMD3100 or vehicle in the growth medium for 72 h. SDF-1α (4 ng/ml) was added at the same time. The cells were stained with the Kwik Diff Stains kit (scale bar, 50 μm). **G** SKRT cells were similarly grown on coverslips and treated with AMD3100 or vehicle in the growth medium for 48 h. After serum starvation overnight, the cells received SDF-1α (100 ng/ml) stimulation for the times indicated and were fixed and permeabilized. CXCR4 was detected with mouse antihuman CXCR4 primary antibody (R&D, Minneapolis, MN) and the Alexa Fluor 488-conjugated goat anti-mouse secondary antibody (green). Nuclei were stained with DAPI (blue). Microscopic images were captured by a multiphoton confocal laser scanning microscope (“Materials and methods” section). Arrows indicate CXCR4 nuclear translocation, white triangles indicate binucleated cells, and yellow triangles indicate giant multinucleated cells

Our studies above demonstrated that the CXCR4 antagonist AMD3100 inhibits proliferation and survival of HER2 + breast cancer cells with primary or acquired trastuzumab resistance. To further discern the mechanism of these effects, we performed functional proteomic analyses. BTRT cells grown in Matrigel 3D culture were treated with vehicle, AMD3100, and/or trastuzumab. Cell lysis was analyzed using RPPA with 484 antibodies (Additional file 9: Table S1). Unsupervised hierarchical clustering showed that AMD3100 monotherapy and combined therapy with trastuzumab formed a cluster at the bottom of the dendrogram (Additional file 3: Fig. S3). As expected, trastuzumab monotherapy did not result in a distinct cluster but formed a cluster with the vehicle, likely because cells had adapted to continuous exposure to trastuzumab. Figure 5A shows an enlarged image of the left part of the panel, showing the significant difference between the two main clusters.

As expected, targeting CXCR4 with AMD3100 inhibited downstream signaling pathways of GPCR, including the MAPK pathway, as indicated by decreased levels of phosphorylation of ERK1/2, p90RSK, p70RSK, S6, and c-Jun, and the PI3K-AKT-mTOR pathway, as shown by decreased phosphorylation of NF-κB, GSK3α/ꞵ, mTOR, 4EBP1, YB-1, and Rb. AMD3100 also reduced the molecules that we demonstrated upregulation in the trastuzumab-resistant breast cancer cells comparing their parental cells, including ERα, Notch3, IGFBP2, and dual specificity phosphatase 4 (DUSP4), which contribute to cancer formation and progression or resistance to anti-HER2 therapy or chemotherapy [45–47]. Intriguingly, AMD3100 suppressed many regulators of the G2/M phases of the cell cycle, particularly, those involved in the G2-M transition checkpoints, as indicated by downregulation of PLK1, FoxM1, Wee1, Myt1, CDC25C, eEF2K, cyclin B1, and reduced the phosphorylation of CDK1, Rb, 4EBP-1, and S6 (Fig. 5A, Additional file 4: Fig. S4). The addition of trastuzumab to AMD3100 increased the inhibitory effect on PLK1, CDK1, and phosphorylation of CDK1 and S6 but did not further increase the inhibitory effect on P-Rb (S807/S811), eEF2K, FoxM1, and RRM2; even reversed the inhibition of AMD3100 on Cyclin B1. The RPPA data were confirmed with western blot analysis (Fig. 5B, Additional file 5: Fig. S5). Taking together, the results suggest that AMD3100 functions at the CXCR4-high expression G2/M phases.

The results from molecular analysis led us to investigate whether targeting CXCR4 affects cell division. Cell cycle analysis showed that AMD3100 dose-dependently increased the number of cells in the G2/M phases in BTRT (Fig. 5C, D) and SKRT cells (Fig. 5C, E). When the AMD3100-treated SKRT cells were analyzed using flow cytometry, a group of cells was automatically identified as doublets, which led us to examine the cell morphology using a modified Wright–Giemsa stain. As expected, AMD3100 induced significant morphologic changes, as indicated by binucleated or giant multinucleated cells (Fig. 5F), which were likely identified as doublets by flow cytometry or were filtered before upload.

We next verified the function of AMD3100 using fluorescence confocal microscopy. SKRT cells were treated with or without AMD3100 and followed by stimulation with SDF-1α. Under the basal condition (without treatment and stimulation), CXCR4 was located in the cell membrane and cytoplasm. Stimulation with SDF-1α induced CXCR4 intracellular trafficking and endocytosis. CXCR4 translocated toward the nuclei or entered the nuclei after the stimulation for 15 min (Fig. 5G), and returned to normal in 30 min (Additional file 6: Fig. S6). AMD3100 dose-dependently induced obvious morphologic changes, with binucleated and giant multinucleated cells, and inhibited CXCR4 nuclear translocation (Fig. 5G, right panels).

Taken together, these results showed that targeting CXCR4 with AMD3100 arrests cell division by inhibiting the mediators of G2-M transition and mitosis, leading to mitotic catastrophe.

### Combined targeting of CXCR4 and docetaxel synergistically inhibits trastuzumab-resistant tumor cell growth in vitro and significantly improves the inhibitory efficacy in vivo

**Fig. 6:**
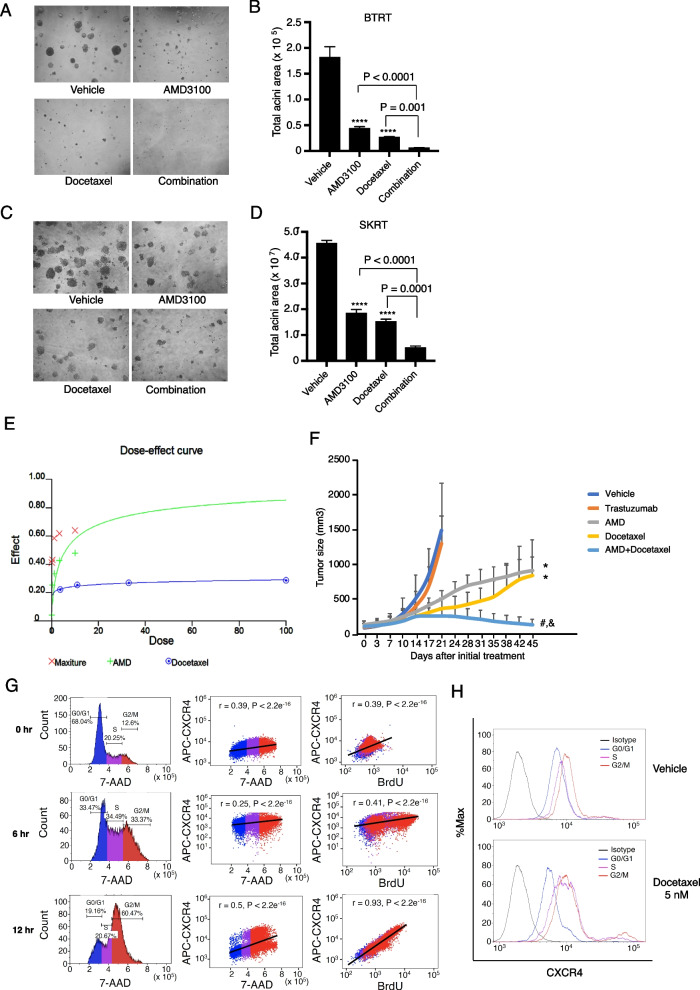
Effect of combined treatment with AMD3100 and docetaxel on acquired trastuzumab-resistant breast tumor growth. **A**, **C** BTRT or SKRT cells were grown in 3D Matrigel culture and treated with AMD3100 (5 µM), docetaxel (10 nM), or the combination. SDF-1α (4 ng/ml) was added at the same time. Photographs were taken on day 9. **B**, **D** Quantitative analysis of total acini area was performed using AlphaView SA, and the data were analyzed using one-way ANOVA. The data are reported as mean ± SD of triplicates, representing two independent experiments (*P < 0.0001 compared with vehicle). **E** BTRT cells grown in 3D Matrigel were treated with serial doses of AMD3100 (AMD) and/or serial doses of docetaxel. SDF-1α (4 ng/ml) was added at the same time. The combined effect of AMD3100 and docetaxel on acini growth was analyzed using CalcuSyn Dose Effect Analyzer. **F** HR6 cells, derived from BT474 cells and exhibiting in vivo acquired trastuzumab resistance, were implanted into the mammary fat pad of female athymic nude mice. When the tumor size reached 100 mm, the mice were randomized to treatment with vehicle, trastuzumab, AMD3100, docetaxel, or different combinations. Tumor volume was calculated using the formula *V* = l*w*^2^/2. Data were analyzed using two-way ANOVA (*P < 0.0001 compared with vehicle, ^#^*P* < 0.0001 compared with AMD3100 alone, ^&^ *P* < 0.0001 compared with docetaxel alone). **G** BTRT cells were treated with docetaxel (5 nM) followed by BrdU pulse. Immunofluorescent staining for CXCR4, BrdU, and 7-AAD was performed and followed by flow cytometry analysis (“Materials and methods” section). The correlation between CXCR4 and 7-AAD or BrdU was analyzed using Pearson r coefficients. **H** BTRT cells were treated with docetaxel or vehicle. Dynamic expression of CXCR4 with cell cycle progression in BTRT cells was detected by flow cytometry

AMD3100 prolonged the cell cycle and slowed down cell growth but did not completely block the G2/M phases. Clinically, chemotherapy is a fundamental component of combined therapies for advanced HER2 + breast cancer except as maintenance following induction therapy [48]. To investigate whether adding the CXCR4 inhibitor AMD3100 to chemotherapy improves efficacy, and which chemotherapy reagents produce the best combinatorial effect, we tested the combination of AMD3100 with cisplatin, carboplatin, and docetaxel. Treatment with AMD3100 or docetaxel inhibited BTRT cell growth in 3D Matrigel culture (*P* < 0.0001 compared with the vehicle). However, the combination of AMD3100 and docetaxel significantly increased the inhibitory effects compared with either drug alone, as indicated by almost completely inhibited acini growth (*P* < 0.0001 compared with AMD3100 alone, *P* < 0.001 compared with docetaxel alone; Fig. 6A, B). The inhibitory effects exhibited a similar pattern in SKRT cells (Fig. 6C, D). We next treated BTRT cells with serial doses of AMD3100 (AMD) and/or docetaxel, followed by synergy analyses. The synergy analyses' dose–effect curve (Fig. 6E) and combination indices (Table 1) indicated synergistic interactions. However, the combination of cisplatin and AMD3100 did not increase their inhibitory effects on cell growth of BTRT (Additional file 7: Fig. S7A, B) or SKRT (Additional file 7: Fig. S7C, D) in Matrigel, even had the opposite effect. The findings were recapitulated using carboplatin to replace cisplatin on BTRT cells (Additional file 7: Fig. S7E, F) and SKRT cells (Additional file 7: Fig. S7G, H).

**Table 1:** Combination index

AMD3100 (µM)	Docetaxel (nM)	CI
0.12	3.7	0.285
0.37	3.7	0.48
1.1	3.7	0.494
3.3	3.7	0.556
10	3.7	0.575
0.12	11	0.409
0.37	11	0.516
1.1	11	0.585
3.3	11	0.605
10	11	0.633
0.12	33	0.457
0.37	33	0.498
1.1	33	0.586
3.3	33	0.609
10	33	0.642
0.12	100	0.413
0.37	100	0.432
1.1	100	0.586
3.3	100	0.619
10	100	0.64

To verify our findings in vivo, we used HR6, an acquired trastuzumab-resistant xenograft model, which was derived from BT474 cells and created by trastuzumab challenge in athymic nude mice [12]. To confirm trastuzumab resistance, we transplanted the HR6 cells into the mammary fat pad of athymic nude mice. The BT-T cells, derived from parental BT474 cells and remaining sensitive to trastuzumab in athymic nude mice, were used as a control. After the tumor size reached 100 mm, all mice were treated with trastuzumab (“Materials and methods” section in detail). Trastuzumab inhibited xenograft growth of BT-T but not HR6 (*P* < 0.0001, Additional file 8: Fig. S8A). Comparing the primary control cells, HR6 exhibited increased CXCR4 expression, but no significant difference in HER2 expression (Additional file 8: Fig. S8B). We next established HR6 xenografts using the same method. The mice with tumor burden were randomly assigned to treatment with vehicle, trastuzumab, AMD3100, docetaxel, or different combinations (Fig. 6F). As expected, trastuzumab did not show an inhibitory effect. AMD3100 or docetaxel monotherapy significantly inhibited the growth of the xenografts (*P* < 0.0001 compared with vehicle). However, the combination of AMD3100 and docetaxel further induced tumor regression (*P* < 0.0001 compared with AMD3100 or docetaxel alone). The addition of trastuzumab to AMD3100/docetaxel tended to increase the inhibitory effect, but the difference was not significant, suggesting that after long-term exposure to trastuzumab, the tumor cells adapted to the drug. We next explored the effects of treatments on the mediators of G2-M transition in vivo. AMD3100 alone or combined with docetaxel suppressed the mediators of the G2-M transition, as indicated by the downregulation of Myt1, FoxM1, cyclin B1, eEF2K, and reduced the phosphorylation of CDK1 and S6 (Additional file 8: Fig. S8C).

Taken together, these results indicated that combined targeting CXCR4 with AMD3100 and docetaxel is a potential novel combination therapy for HER2 + breast cancer with trastuzumab resistance.

### AMD3100 synergistically interacts with docetaxel by suppressing docetaxel-induced CXCR4 upregulation in trastuzumab-resistant breast cancer

We next explored the mechanism of the synergistic interactions of AMD3100 and docetaxel. After being treated with docetaxel, BTRT cells received BrdU pulse, and then, dynamic expression of CXCR4 in the cell cycle phases was measured using flow cytometry (“Materials and methods” section; Fig. 3J). The microtubule inhibitor docetaxel arrested the cells in the M phase (Fig. 6G, left panel). CXCR4 expression levels markedly increased from the S phase and reached a peak in the G2/M phases (Fig. 6G, H). CXCR4 protein levels reached their highest point at 12 h after treatment and were highly correlated with BrdU (*r* = 0.93, *P* < 2.2e^−18^; Fig. 6G, right panel). These results indicate that CXCR4 upregulation is a response of the cells to docetaxel, possibly a self-protective mechanism. The addition of AMD3100 suppressed the response to docetaxel, thus synergistically inhibiting tumor cell growth.

### CXCR4 is upregulated in residual diseases than primary breast tumors

**Fig. 7:**
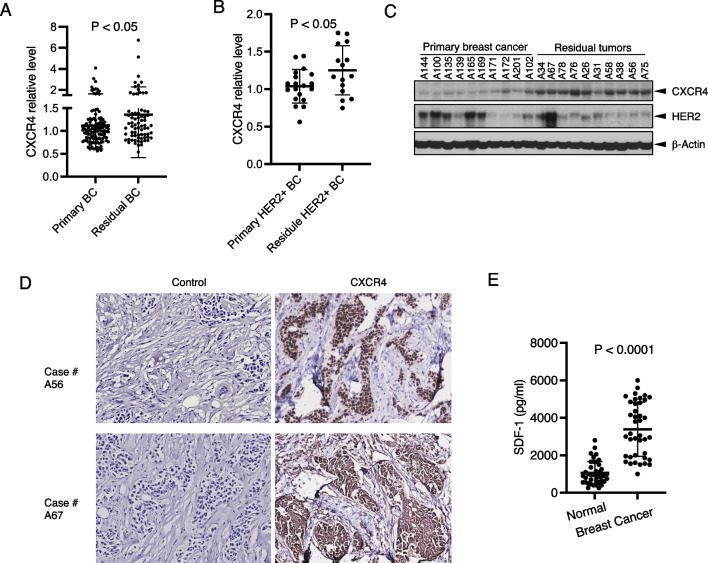
Upregulation of CXCR4 in residual disease of breast cancer. **A** CXCR4 protein in 72 residual tumor tissues and 112 primary tumor samples from breast cancer patients was analyzed using RPPA. **B** CXCR4 protein in residual HER2 + breast cancer after treatment with trastuzumab and chemotherapy compared with primary HER2 + breast cancer. **C** CXCR4 expression in breast cancer tissues was confirmed with western blot. **D** Representative human HER2 + breast cancer residual breast tumor tissue samples with immunohistochemical staining. Original magnification, 200x. **E** Serum samples from breast cancer patients or healthy women were measured for SDF-1α with ELISA. Data were analyzed using *t*-test

To investigate the role of CXCR4 in trastuzumab resistance in breast cancer patients, using RPPA, we analyzed CXCR4 expression in fresh-frozen tumor tissues from 112 untreated patients (primary breast tumor tissues) and 72 patients who received neoadjuvant therapy before surgery (residual tumor tissues) that we had collected and some of which were used in our previous studies [41], among them, several patients with HER2 + breast cancer received Herceptin and Taxol (paclitaxel), or Taxotere (docetaxel). CXCR4 expression was increased in the residual disease samples compared with the primary tumors (*P* < 0.05; Fig. 7A). In the cohort tested, in total 34 samples were HER2+, including 19 primary tumor tissues and 15 residual tumor samples. Compared with the primary tumor tissues, the residual disease samples exhibited higher CXCR4 protein after treatment with trastuzumab and chemotherapy (*P* < 0.05) (Fig. 7B). To confirm the RPPA data, we analyzed additional HER2 + samples with western blot for CXCR4 expression (Fig. 7C). To examine the cellular and subcellular distribution of CXCR4 expression in HER2 + breast cancer residual tissues, we performed immunohistochemical staining and confirmed CXCR4 expression in tumor cells (Fig. 7D). Intriguingly, CXCR4 expression in tumor cells exhibited different patterns. Here we show two cases to represent the differences. Case 1 in Fig. 7D shows increased CXCR4 mainly in nuclei, while Case 2 in Fig. 7D shows increased CXCR4 mainly in the cell membrane and cytoplasm. The underlying biological and clinical significance needs to be further explored by increasing the number of cases. Taken together, the evidence supports the contribution of CXCR4 to drug resistance.

We also measured circulating SDF-1α in breast cancer patients using ELISA. Serum SDF-1α levels in breast cancer patients were 1005–6005 pg/ml (average 3393 pg/ml). Its range in the healthy women controls was 255–2805 pg/ml (average 1060 pg). The results demonstrated that SDF-1α was significantly higher in breast cancer patients compared to the healthy controls (*P* < 0.0001) (Fig. 7E). The natural SDF-1α level in patients informed the concentration of SDF-1α (4 ng/ml) chosen for the experiments of proliferation, survival, and signaling transduction.

## Discussion

Trastuzumab is the first approved anti-HER2 targeted therapy in breast cancer. Although several HER2-directed therapies have been developed subsequently, such as pertuzumab, lapatinib, neratinib, T-DM1, and trastuzumab deruxtecan, trastuzumab still serves as the backbone of HER2-targeted therapy. However, resistance to trastuzumab remains a clinical challenge. Amplification or mutation of HER2 and upregulation of downstream PI3K/AKT and MAPK pathway are usually proposed as the mechanism of drug resistance [9–17]. We previously reported that CXCR4 is involved as a driver of trastuzumab resistance in HER2 + breast cancer cells [18–20] with an unknown mechanism. In the current study, we demonstrated that the increased CXCR4 expression in trastuzumab-resistant tumor cells is associated with cell cycle progression and reaches a peak in the G2/M phases, contributing to cell proliferation and survival. To our knowledge, this is the first study to demonstrate that CXCR4 plays a role in cell cycle progression in cancer cells. Intriguingly, a similar phenomenon was reported in germinal center B cells [49]. SDF1α/CXCR4 axis plays a role in tumor progression with the proposed mechanism that the binding of SDF-1α to its GPCR CXCR4 activates the downstream MAPK, PI3K pathways, which lead to cell proliferation, survival, chemotaxis, and transcription of gene expression [26–32]. Stimulation with SDF-1α induced CXCR4 nuclear translocation. Mitotic mediators usually translocate into the nucleus for cell division [50]. Our functional proteomic analysis revealed that targeting CXCR4 with its antagonist AMD3100 downregulated the G2-M transition-associated proteins that are strictly required to complete mitosis, including PLK1, FoxM1, Wee1, Myt1, CDC25C, and cyclin B1. Consistently, flow cytometry analysis indicated G2-M arrest, and imaging analysis showed SDF-1α-induced CXCR4 nuclear translocation was arrested, leading to mitotic catastrophe, characterized by the formation of binucleated or multinucleated large cells. The molecular changes and cellular biological phenomena induced by AMD3100 and the characteristics of CXCR4's dynamic expression with cell cycle progression corroborate each other. Taken together, our results suggest the underlying mechanism of targeting CXCR4 with AMD3100 to inhibit trastuzumab-resistant tumor cell growth operates via mitotic catastrophe and mitotic death. However, detailed interrogation is needed to explain how CXCR4 regulates mitosis to promotes cell proliferation and survival. Interestingly, a recent study demonstrated that HER2 promotes tumorigenesis by direct regulation of cell mitotic progression through activating Shc1–SHCBP1–PLK1–MISP axis. Hyper activating Shc1–SHCBP1–PLK1–MISP axis impairs sensitivity of HER2-positive gastric cancer to trastuzumab [51]. However, it is unknown whether CXCR4 is the upstream of the pathway. In addition, a systematic study on how long-term trastuzumab challenge leads to CXCR4 upregulation is required in the future.

Using functional proteomics analysis, we examined CXCR4 protein expression in 184 fresh-frozen breast tumor tissues, including 34 HER2+, from breast cancer patients with or without chemotherapy and/or trastuzumab treatment, consistent with our findings from the cell lines, comparing the primary tumors, CXCR4 expression significantly increased in the residual tumor tissues, suggesting that CXCR4 can be a biomarker to predict the drug resistance. Supporting our findings, in a very recent report, a retrospective clinical study investigated CXCR4 expression in 62 formalin-fixed paraffin-embedded tissue specimens using RT-qPCR and immunohistochemistry and found upregulation of CXCR4 in trastuzumab-treated samples. High CXCR4 expression was associated with recurrence [21]. Teams from different countries using different methods showed CXCR4 upregulation in breast cancer with trastuzumab resistance.

Breast cancer cells cultured in 3D showed different responses to chemotherapies than those observed in cells cultured in 2D [52]. Given the known role of CXCR4 signaling in the tumor/immune microenvironment, to better understand how CXCR4 signaling contributes to trastuzumab resistance, it should be noted that we used 3D co-cultures or 3D Matrigel culture with the supplement of SDF-1α. Regarding the concentration of recombinant SDF-1α used for studies, incubation of cells with high concentration (100 nM = 1160 ng/ml) for 3 h or more induces significant degradation of CXCR4 [53], while stimulation with 100 ng/ml of SDF-1α for 1 h did not decrease CXCR4 expression [31]. In the present study, we used the known concentration of SDF-1α in breast cancer patients (4 ng/ml) for long-term (a few days) experiments. For the short-term experiments (1 h or less), we used 100 ng/ml. Recapitulating the stromal and immune environment in our model systems demonstrated an enhanced impact of our strategy as expected.

Studies showed that CXCR4 is involved in resistance to chemotherapies, including paclitaxel, a taxane similar to docetaxel, in ovarian cancer [54] and ER+ or triple-negative breast cancer cell lines [55]. Our study, as the first, demonstrated that targeting CXCR4 synergizes with docetaxel in HER2 + breast cancer with trastuzumab resistance. We also identified the direct mechanism that CXCR4 upregulation is a response of the tumor cells to docetaxel, and AMD3100 blocks the protective adaptation.

## Conclusion

The present study provided preclinical evidence that targeting CXCR4 abrogates trastuzumab resistance by blocking cell cycle progression and synergizes with docetaxel in trastuzumab-resistant breast cancer treatment. Our findings therefore demonstrated that CXCR4 is a promising therapeutic target and a predictive biomarker in HER2 + breast cancer with trastuzumab resistance. Our next goal is a biomarker-driven prospective trial of trastuzumab plus CXCR4 inhibitor either with or without docetaxel in patients who have exhibited resistance to trastuzumab.

## Supplementary Information

**Additional file 1**. **Figure S1**: Illustration of 3D co-culture. BTRT or SKRT cells were co-cultured with BCAFs in 96-well “U”-bottomed unattached plates. The spheres were treated with AMD3100 on day 3. For three line-co-culture, PBMCs were seeded on day 5, followed by treatment with trastuzumab. Cell viability was detected with CellTiterGlo.

**Additional file 2. Figure S2**. Effect of AMD3100 on cell growth in 3D co-culture. BTRT or SKRT were co-cultured with BCAFs in 96-well “U”-bottomed unattached plates and treated with AMD3100. The dynamic change of the spheres was monitored and photographed.

**Additional file 3**. **Figure S3**. BTRT cells grown in 3D Matrigel culture were treated with vehicle, AMD3100, trastuzumab, or their combination. SDF-1αwas added at the same time. Cell lysates were subject to RPPA

**Additional file 4**. **Figure S4**. Effect of AMD3100 on pathways. BTRT cells grown in 3D Matrigel culture were treated with vehicle, AMD3100, trastuzumab, or their combination. Cell lysates were subject to RPPA. A portion of the RPPA data were further analyzed with one-way ANOVA.

**Additional file 5**. **Figure S5**. Western blot. BTRT cells grown in 3D Matrigel culture were treated with vehicle, AMD3100, trastuzumab, or their combination. SDF-1α was added at the same time. A polypeptide that was designed as a CXCR4 antagonist but did not show biological function in the HER2+ breast cancer cells was used as a negative control.

**Additional file 6**. **Figure S6**: Translocation of CXCR4 and changes of cell morphology induced by SDF-1a. SKRT cells were grown on coverslips. After serum starvation overnight, the cells received stimulation with SDF-1a for the times indicated and were fixed and permeabilized. CXCR4 was detected with mouse anti-human CXCR4 primary antibody and the Alexa Fluor 488-conjugated goat anti-mouse secondary antibody. Nuclei were stained with DAPI. Microscopic images were captured by a multiphoton confocal laser scanning microscope. Arrows indicate CXCR4 nuclear translocation.

**Additional file 7**. **Figure S7**: Effect of combination of AMD3100 and cisplatin or carboplatin on trastuzumab-resistant breast cancer cell growth. BTRT or SKRT cells were grown in 3D Matrigel culture and treated with AMD3100/cisplatin, or AMD3100/carboplatin. Photographs were taken on day 11. Quantitative analysis of the total acini area was performed using AlphaView SA, and the data were analyzed using one-way ANOVA (B, D, F, H: *, P < 0.0001 vs. Vehicle.

**Additional file 8**. **Figure S8**: Characteristic of the trastuzumab-resistant mouse model and effect of combined treatments on cell signaling. A five-week-old female athymic nude mice were implanted with 0.36 mg, 90-day release, 17beta-estradiol pellets. Three days later, in total 5x10^6^ BT-T or HR6 cells in 150 μl growth factor reduced Matrigel and PBS were orthotopically injected. Once tumors reached a volume ~100 mm3, the mice were randomly grouped and received treatment with trastuzumab. Tumor sizes were measured with calipers twice weekly. Tumor volume was calculated with the formula V = lw2/2. Differences in tumor volume between groups were analyzed using two-way ANOVA. B HR-6 or BT-T tumor cells were grown in 3D Matrigel culture for 6 days, and cell lysates were analyzed with western blot. C HR6 cells were implanted into the mammary fat pad of female athymic nude mice. The mice with tumor burden were randomized to treatment with vehicle, trastuzumab, AMD3100, docetaxel, or combinations as indicated. The xenograft tumor lysates were analyzed with western blot.

**Additional file 9**. **Table S1**: Antibodies used for RPPA and western blot.

## Data Availability

The data generated and/or analyzed during this study are available from the corresponding author on reasonable request.

## Abbreviations

HER2+: Human epidermal growth factor receptor 2 positive
PI3K: Phosphatidylinositide 3 kinase
IGF-1R: The insulin-like growth factor-I receptor
EGFR: Epidermal growth factor receptor
CXCR4: C-X-C motif chemokine receptor 4
CXCL12: C-X-C motif chemokine ligand 12
SDF-1: Stromal cell-derived factor-1
3D: Three-dimensional
BrdU: 5′-Bromo-2′-deoxyuridine
FBS: Fetal bovine serum
7-AAD: 7-Amino-actinomycin D
DAPI: 4′, 6-Diamidino-2-phenylindole
ELISA: Enzyme-linked immunosorbent assay
CFDA, SE: 5- (and 6)-Carboxyfluorescein diacetate, succinimidyl ester
EDTA: Ethylenediaminetetraacetic acid
ADCC: Antibody-dependent cellular cytotoxicity
MAPK: Mitogen-activated protein kinase
PBMC: Peripheral blood mononuclear cell
ER: Estrogen receptor
BCAF: Breast cancer-associated fibroblast
RPPA: Reverse phase protein array
ANOVA: Analysis of variance
PBS: Phosphate-buffered saline
GPCR: G protein-coupled receptor
ER+: Estrogen receptor positive
BTRT: BT474-derived trastuzumab-resistant cells
SKRT: SKBR3-derived trastuzumab-resistant cells
