# Supplementary material for: Targeting CXCR4 abrogates resistance to trastuzumab by blocking cell cycle progression and synergizes with docetaxel in breast cancer treatment
Source: Breast Cancer Res. 2023 Jun 6;25:62. doi: 10.1186/s13058-023-01665-w (PMC10245436; doi:10.1186/s13058-023-01665-w)
Supplement: Supplementary file 1 — Additional file 1. Figure S1: Illustration of 3D co-culture. BTRT or SKRT cells were co-cultured with BCAFs in 96-well “U”-bottomed unattached plates. The spheres were treated with AMD3100 on day 3. For three line-co-culture, PBMCs were seeded on day 5, followed by treatment with trastuzumab. Cell viability was detected with CellTiterGlo. [file 13058_2023_1665_MOESM1_ESM.pdf]

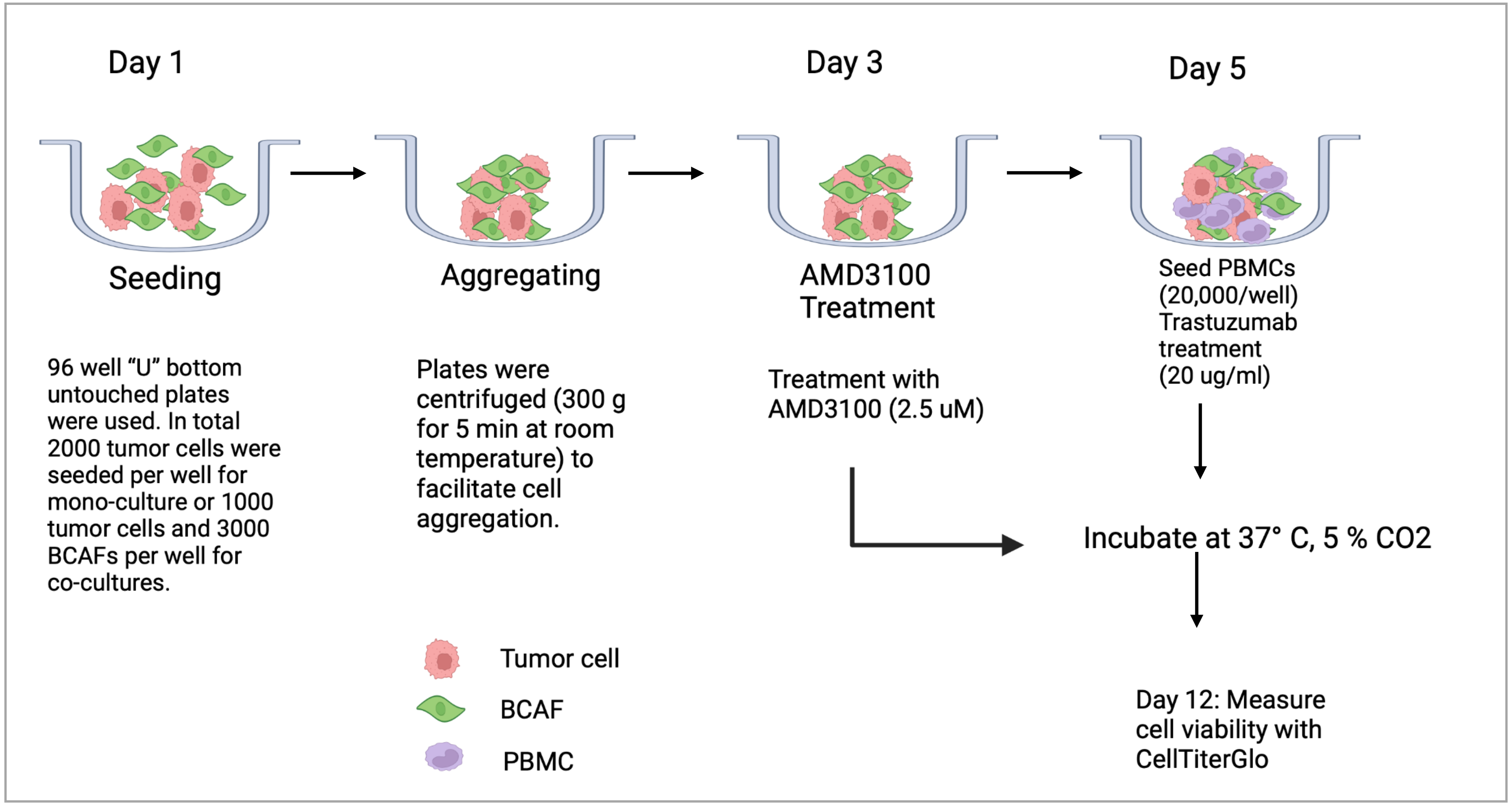

Fig. S1. **Illustration of 3D co-culture.** BTRT or SKRT cells were cocultured with BCAFs in 96 well “U” bottom unattached plates. The spheres were treated with AMD3100 (2.5 µM) on day 3. For three line-co-culture, PBMCs were seeded on day 5, followed by treatment with trastuzumab (20 ug/ml). Cell viability was detected with CellTiterGlo.
