# Supplementary material for: Targeting CXCR4 abrogates resistance to trastuzumab by blocking cell cycle progression and synergizes with docetaxel in breast cancer treatment
Source: Breast Cancer Res. 2023 Jun 6;25:62. doi: 10.1186/s13058-023-01665-w (PMC10245436; doi:10.1186/s13058-023-01665-w)
Supplement: Supplementary file 2 — Additional file 2. Figure S2. Effect of AMD3100 on cell growth in 3D co-culture. BTRT or SKRT were co-cultured with BCAFs in 96-well “U”-bottomed unattached plates and treated with AMD3100. The dynamic change of the spheres was monitored and photographed. [file 13058_2023_1665_MOESM2_ESM.pdf]

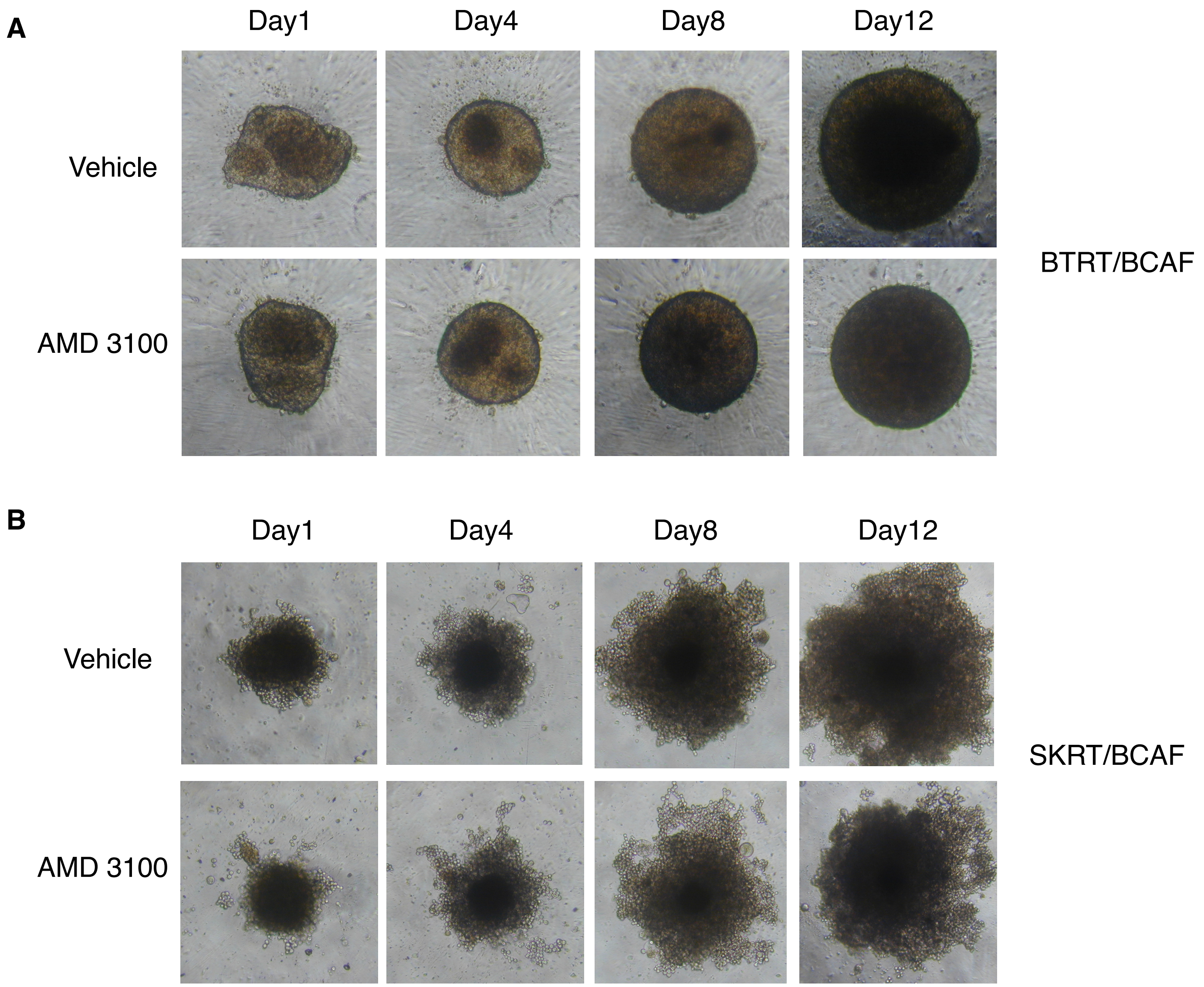

Fig. S2. **Effect of AMD3100 on cell growth in 3D coculture.** BTRT (A) or SKRT (B) were cocultured with BCAFs in 96 well "U" bottom unattached plates and treated with AMD3100 (2.5 μM) (Material and Methods). The dynamic change of the spheres was monitored and photographed.
