# Supplementary material for: Targeting CXCR4 abrogates resistance to trastuzumab by blocking cell cycle progression and synergizes with docetaxel in breast cancer treatment
Source: Breast Cancer Res. 2023 Jun 6;25:62. doi: 10.1186/s13058-023-01665-w (PMC10245436; doi:10.1186/s13058-023-01665-w)
Supplement: Supplementary file 4 — Additional file 4. Figure S4. Effect of AMD3100 on pathways. BTRT cells grown in 3D Matrigel culture were treated with vehicle, AMD3100, trastuzumab, or their combination. Cell lysates were subject to RPPA. A portion of the RPPA data were further analyzed with one-way ANOVA. [file 13058_2023_1665_MOESM4_ESM.pdf]

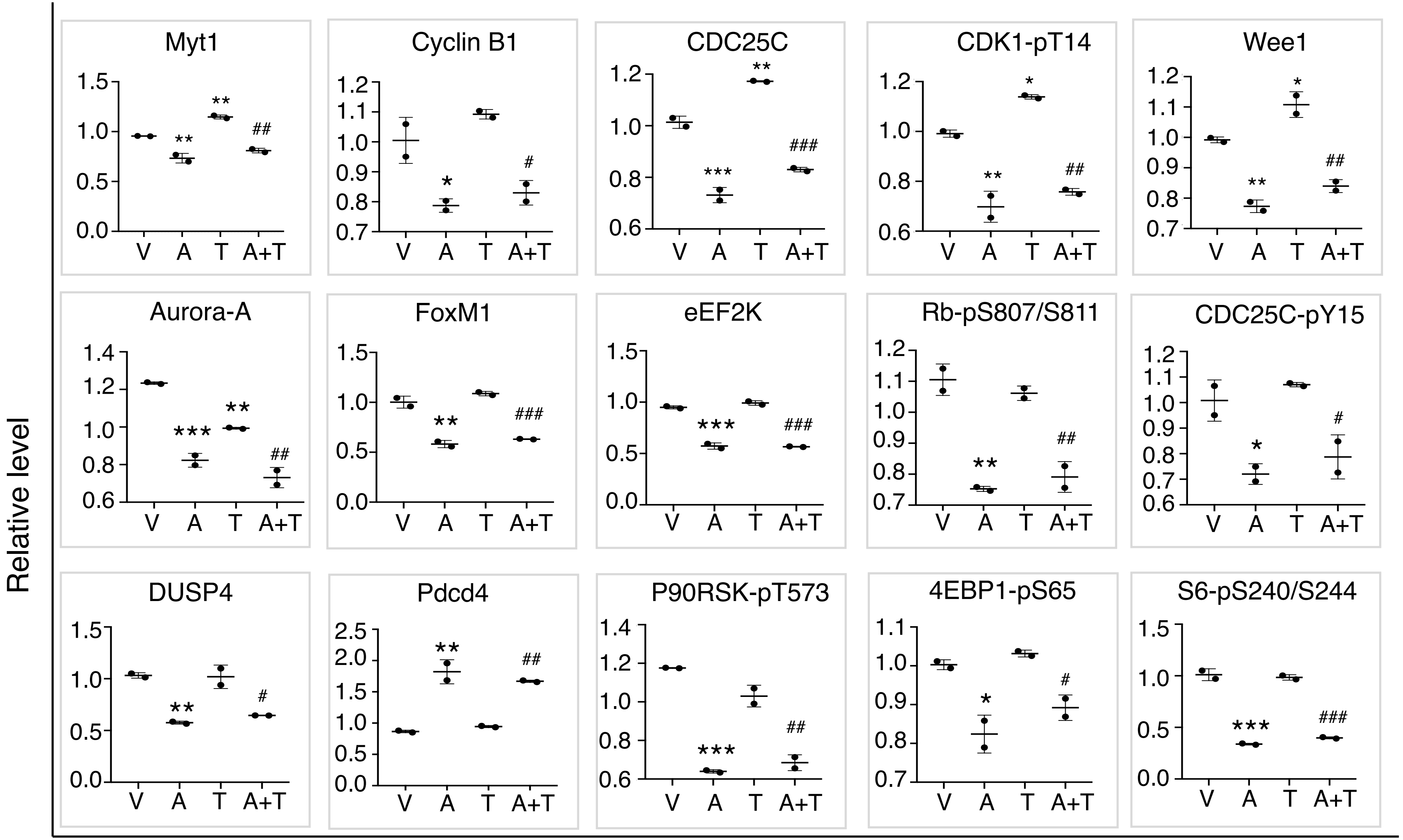

Fig. S4. **Effect of AMD3100 on pathways.** BTRT cells grown in 3D Matrigel culture were treated with vehicle (V), AMD3100 (A; 10μM), trastuzumab (T; 20 μg/ml), or their combination (A+T). Cell lysates were subject to RPPA (Materials and Methods, Fig. 5A). A portion of the RPPA data were further analyzed with one-way ANOVA (mean ± SD; P value based on log2 data: *$P < 0.05$, **$P < 0.01$, ***$P < 0.001$ compared with the vehicle; #$P < 0.05$, ##$P < 0.01$, ###$P < 0.001$ compared with trastuzumab).
