# Supplementary material for: Targeting CXCR4 abrogates resistance to trastuzumab by blocking cell cycle progression and synergizes with docetaxel in breast cancer treatment
Source: Breast Cancer Res. 2023 Jun 6;25:62. doi: 10.1186/s13058-023-01665-w (PMC10245436; doi:10.1186/s13058-023-01665-w)
Supplement: Supplementary file 5 — Additional file 5. Figure S5. Western blot. BTRT cells grown in 3D Matrigel culture were treated with vehicle, AMD3100, trastuzumab, or their combination. SDF-1α was added at the same time. A polypeptide that was designed as a CXCR4 antagonist but did not show biological function in the HER2+ breast cancer cells was used as a negative control. [file 13058_2023_1665_MOESM5_ESM.pdf]

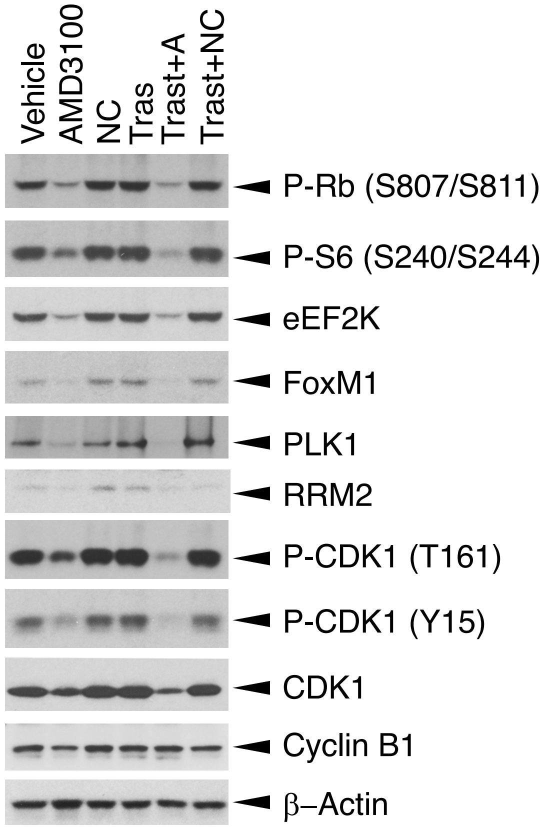

Fig. S5. Western Blot. BTRT cells grown in 3D Matrigel culture were treated with vehicle, AMD3100 (10μM), trastuzumab (20 μg/ml), or their combination. SDF-1α (4 ng/ml) was added at the same time (Material and Methods). A polypeptide that was designed as a CXCR4 antagonist but did not show biological function in the HER2+ breast cancer cells was used as a negative control (NC).
