# Supplementary material for: Targeting CXCR4 abrogates resistance to trastuzumab by blocking cell cycle progression and synergizes with docetaxel in breast cancer treatment
Source: Breast Cancer Res. 2023 Jun 6;25:62. doi: 10.1186/s13058-023-01665-w (PMC10245436; doi:10.1186/s13058-023-01665-w)
Supplement: Supplementary file 3 — Additional file 3. Figure S3. BTRT cells grown in 3D Matrigel culture were treated with vehicle, AMD3100, trastuzumab, or their combination. SDF-1αwas added at the same time. Cell lysates were subject to RPPA [file 13058_2023_1665_MOESM3_ESM.pdf]

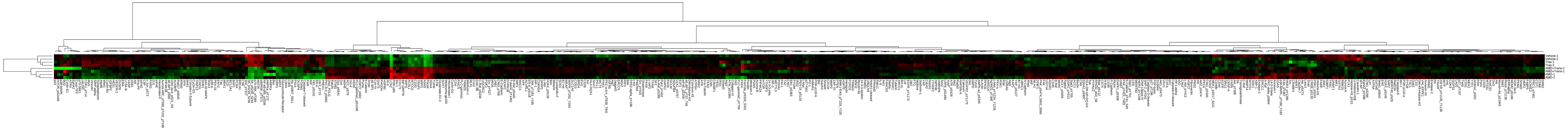
Vehicle-1
Vehicle-2
Tras-1
Tras-2
AMD+Trans-2
AMD+Trans-1
AMD-1
AMD-2
