# Supplementary material for: Targeting CXCR4 abrogates resistance to trastuzumab by blocking cell cycle progression and synergizes with docetaxel in breast cancer treatment
Source: Breast Cancer Res. 2023 Jun 6;25:62. doi: 10.1186/s13058-023-01665-w (PMC10245436; doi:10.1186/s13058-023-01665-w)
Supplement: Supplementary file 7 — Additional file 7. Figure S7: Effect of combination of AMD3100 and cisplatin or carboplatin on trastuzumab-resistant breast cancer cell growth. BTRT or SKRT cells were grown in 3D Matrigel culture and treated with AMD3100/cisplatin, or AMD3100/carboplatin. Photographs were taken on day 11. Quantitative analysis of the total acini area was performed using AlphaView SA, and the data were analyzed using one-way ANOVA (B, D, F, H: *, P < 0.0001 vs. Vehicle. [file 13058_2023_1665_MOESM7_ESM.pdf]

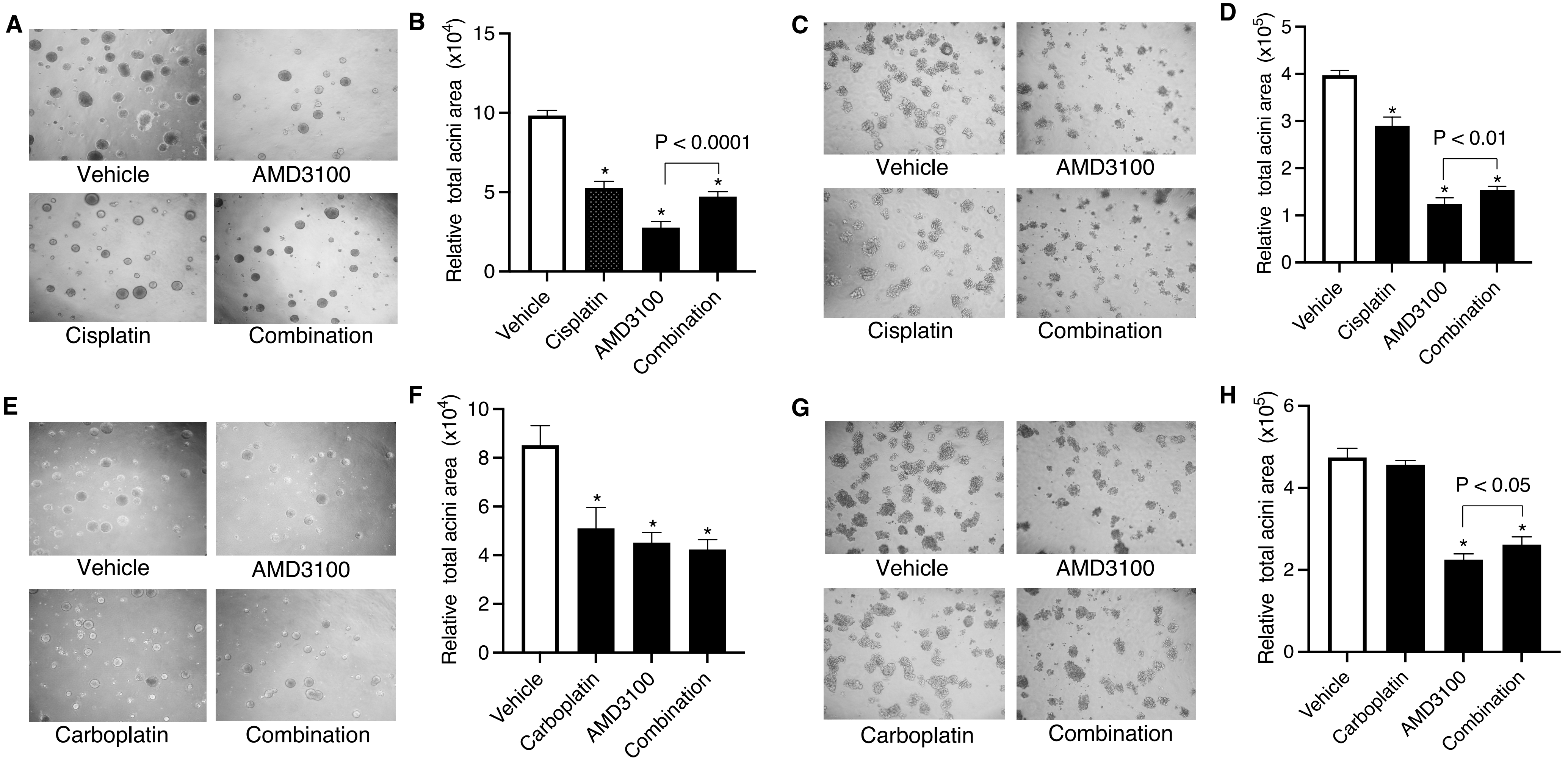

Fig. S7. **Effect of combination of AMD3100 and Cisplatin or carboplatin on trastuzumab-resistant breast cancer cell growth.** BTRT (A, E) or SKRT cells (C, G) were grown in 3D Matrigel culture and treated with AMD3100 (5 µM)/Cisplatin (10 µM) (A, C), or AMD3100 (5 µM)/Carboplatin (10 µM) (E, G). Photographs were taken on day 11. Quantitative analysis of the total acini area was performed using AlphaView SA, and the data were analyzed using one-way ANOVA (B, D, F, H: *, $P < 0.0001$ vs. Vehicle.
