# Supplementary material for: Targeting CXCR4 abrogates resistance to trastuzumab by blocking cell cycle progression and synergizes with docetaxel in breast cancer treatment
Source: Breast Cancer Res. 2023 Jun 6;25:62. doi: 10.1186/s13058-023-01665-w (PMC10245436; doi:10.1186/s13058-023-01665-w)
Supplement: Supplementary file 8 — Additional file 8. Figure S8: Characteristic of the trastuzumab-resistant mouse model and effect of combined treatments on cell signaling. A five-week-old female athymic nude mice were implanted with 0.36 mg, 90-day release, 17beta-estradiol pellets. Three days later, in total 5x10^6^ BT-T or HR6 cells in 150 μl growth factor reduced Matrigel and PBS were orthotopically injected. Once tumors reached a volume ~100 mm3, the mice were randomly grouped and received treatment with trastuzumab. Tumor sizes were measured with calipers twice weekly. Tumor volume was calculated with the formula V = lw2/2. Differences in tumor volume between groups were analyzed using two-way ANOVA. B HR-6 or BT-T tumor cells were grown in 3D Matrigel culture for 6 days, and cell lysates were analyzed with western blot. C HR6 cells were implanted into the mammary fat pad of female athymic nude mice. The mice with tumor burden were randomized to treatment with vehicle, trastuzumab, AMD3100, docetaxel, or combinations as indicated. The xenograft tumor lysates were analyzed with western blot. [file 13058_2023_1665_MOESM8_ESM.pdf]

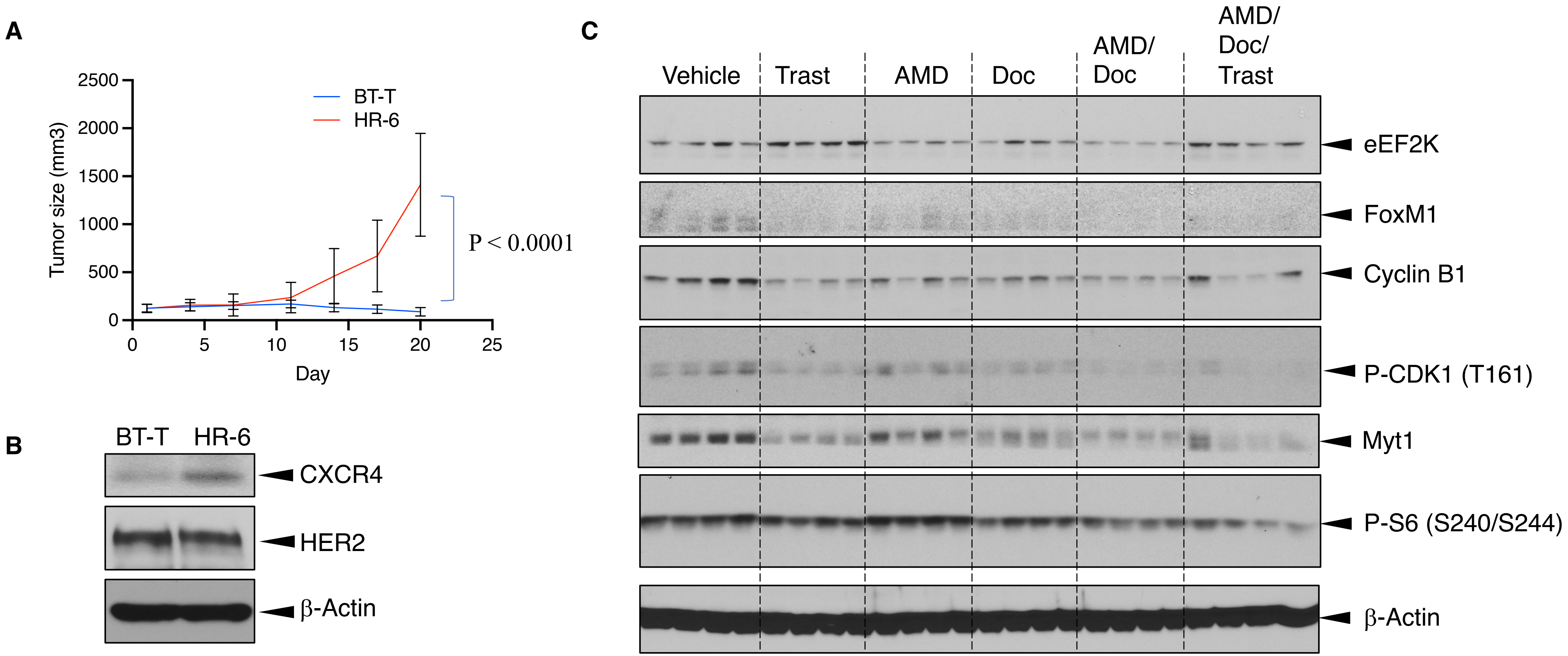

Fig. S8. **Characteristic of the trastuzumab-resistant mouse model and effect of combined treatments on cell signaling. A** Five-week-old female athymic nude mice were implanted with 0.36 mg, 90-day release, 17β-estradiol pellets. Three days later, in total 5×10^6 BT-T or HR6 cells in 150 µl growth factor reduced Matrigel and PBS (1:1) were orthotopically injected. Once tumors reached a volume ~100 mm3, the mice were randomly grouped and received treatment with vehicle, trastuzumab (20 mg/kg, i.p., twice/week). Tumor sizes were measured with calipers twice weekly. Tumor volume was calculated with the formula V = lw2/2. Differences in tumor volume between groups were analyzed using two-way ANOVA. **B** HR-6 or BT-T tumor cells were grown in 3D Matrigel culture for 6 days, and cell lysates were analyzed with Western Blot. **C** HR6 cells were implanted into the mammary fat pad of female athymic nude mice. The mice with tumor burden were randomized to treatment with vehicle, trastuzumab (Trast), AMD3100 (AMD), docetaxel (Doc), or combinations as indicated (In detail see Material and Methods, Fig. 6F). The xenograft tumor lysates were analyzed with Western Blot.
