# Supplementary material for: Targeting CXCR4 abrogates resistance to trastuzumab by blocking cell cycle progression and synergizes with docetaxel in breast cancer treatment
Source: Breast Cancer Res. 2023 Jun 6;25:62. doi: 10.1186/s13058-023-01665-w (PMC10245436; doi:10.1186/s13058-023-01665-w)
Supplement: Supplementary file 6 — Additional file 6. Figure S6: Translocation of CXCR4 and changes of cell morphology induced by SDF-1a. SKRT cells were grown on coverslips. After serum starvation overnight, the cells received stimulation with SDF-1a for the times indicated and were fixed and permeabilized. CXCR4 was detected with mouse anti-human CXCR4 primary antibody and the Alexa Fluor 488-conjugated goat anti-mouse secondary antibody. Nuclei were stained with DAPI. Microscopic images were captured by a multiphoton confocal laser scanning microscope. Arrows indicate CXCR4 nuclear translocation. [file 13058_2023_1665_MOESM6_ESM.pdf]

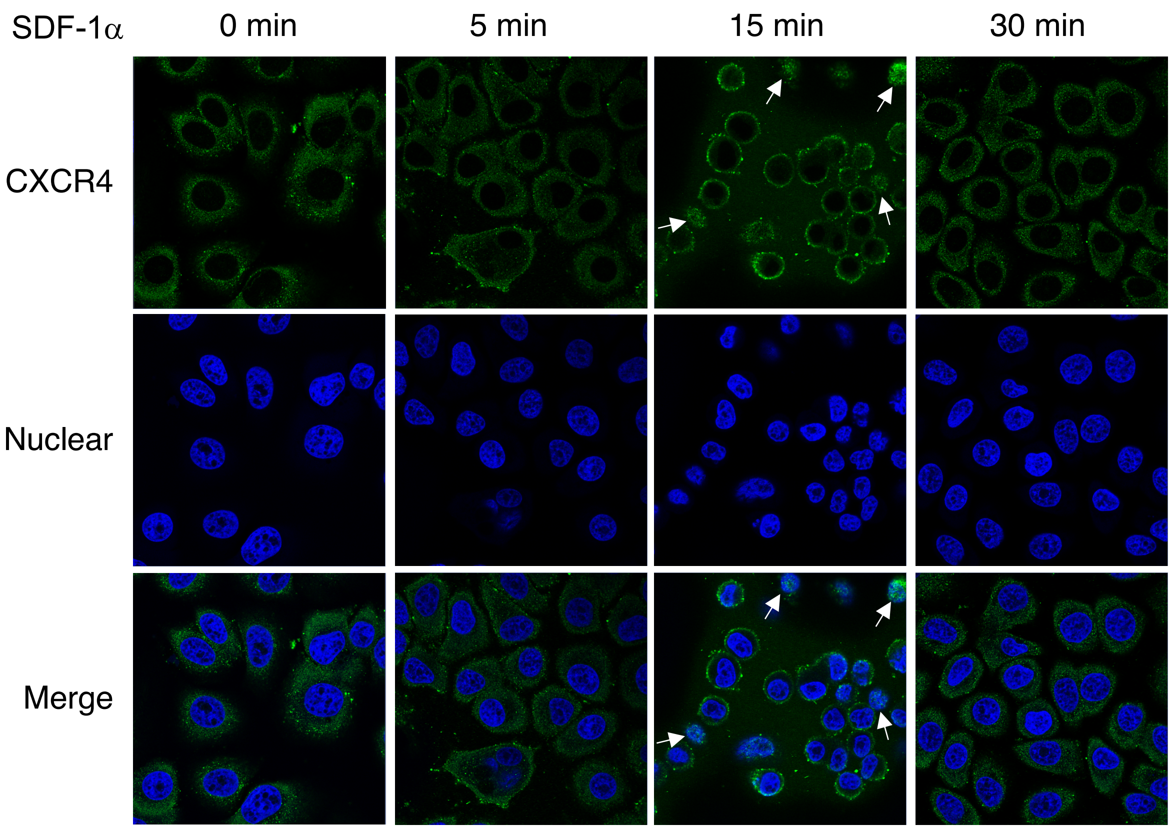

Fig. S6. **Translocation of CXCR4 and changes of cell morphology induced by SDF-1α.** SKRT cells were grown on coverslips. After serum starvation overnight, the cells received stimulation with SDF-1α (100 ng/ml) for the times indicated and were fixed and permeabilized. CXCR4 was detected with mouse anti-human CXCR4 primary antibody (R&D, Minneapolis, MN) and the Alexa Fluor 488–conjugated goat anti-mouse secondary antibody (green). Nuclei were stained with DAPI (blue). Microscopic images were captured by a multiphoton confocal laser-scanning microscope (Materials and Methods). Arrows indicate CXCR4 nuclear translocation.
